# Results from a difference‐in‐differences evaluation of health facility HIV and key population stigma‐reduction interventions in Ghana

**DOI:** 10.1002/jia2.25483

**Published:** 2020-04-23

**Authors:** Laura Nyblade, Nii A Addo, Kyeremeh Atuahene, Nabil Alsoufi, Emma Gyamera, Suzie Jacinthe, Madeline Leonard, Pia Mingkwan, Christin Stewart, Richard Vormawor, John D Kraemer

**Affiliations:** ^1^ Global Health Division Research Triangle Institute (RTI) International Washington DC USA; ^2^ Research Triangle Institute (RTI) International Research Triangle Park NC USA; ^3^ Educational Assessment Research Centre (EARC) Accra Ghana; ^4^ Ghana AIDS Commission Accra Ghana; ^5^ USAID Accra Ghana; ^6^ Department of Health Systems Administration Georgetown University Washington DC USA

**Keywords:** stigma, HIV, health care facilities, health care workers, discrimination, key populations

## Abstract

**Introduction:**

Stigma undermines all aspects of a comprehensive HIV response, as reflected in recent global initiatives for stigma‐reduction. Yet a commensurate response to systematically tackle stigma within country responses has not yet occurred, which may be due to the lack of sufficient evidence documenting evaluated stigma‐reduction interventions. With stigma present in all life spheres, health facilities offer a logical starting point for developing and expanding stigma reduction interventions. This study evaluates the impact of a “total facility” stigma‐reduction intervention on the drivers and manifestations of stigma and discrimination among health facility staff in Ghana.

**Methods:**

We evaluated the impact of a total facility stigma‐reduction intervention by comparing five intervention to five comparable non‐intervention health facilities in Ghana. Interventions began in September 2017. Data collection was in June 2017 and April 2018. The primary outcomes were composite indicators for three stigma drivers, self‐reported stigmatizing avoidance behaviour, and observed discrimination. The principal intervention variable was whether the respondent worked at an intervention or comparison facility. We estimated intervention effects as differences‐in‐differences in each outcome, further adjusted using inverse probability of treatment weighting (IPTW).

**Results:**

We observed favourable intervention effects for all outcome domains except for stigmatizing attitudes. Preferring not to provide services to people living with HIV (PLHIV) or a key population member improved 11.1% more in intervention than comparison facility respondents (95% CI 3.2 to 19.0). Other significant improvements included knowledge of policies to protect against discrimination (difference‐in‐differences = 20.4%; 95% CI 12.7 to 28.0); belief that discrimination would be punished (11.2%; 95% CI 0.2 to 22.3); and knowledge of and belief in the adequacy of infection control policies (17.6%; 95% CI 8.3 to 26.9). Reported observation of stigma and discrimination incidents fell by 7.4 percentage points more among intervention than comparison facility respondents, though only marginally significant in the IPTW‐adjusted model (*p* = 0.06). Respondents at intervention facilities were 19.0% (95% CI 12.2 to 25.8) more likely to report that staff behaviour towards PLHIV had improved over the last year than those at comparison facilities.

**Conclusions:**

These results provide a foundation for scaling up health facility stigma‐reduction within national HIV responses, though they should be accompanied by rigorous implementation science to ensure ongoing learning and adaptation for maximum effectiveness and long‐term impact.

## 1 Introduction

Mounting evidence documents that stigma undermines HIV prevention strategies such as pre‐exposure prophylaxis [1], HIV testing [2, 3, 4], linkage to and retention in care [5, 6, 7], medication adherence [8, 9, 10, 11, 12, 13] and ultimately viral load suppression [2, 14, 15]. Increasingly recognized as a key determinant of health and health inequity [16], stigma is a powerful social process characterized by labelling (distinguishing differences), stereotyping (attributing negative characteristics to the distinguished differences), separation (through physical and social isolation), leading to status loss (social and economic) and discrimination, all occurring in the context of power [17]. There are multiple types of stigma. Experienced stigma is enacted through interpersonal acts of discrimination that can range from verbal and physical abuse to social isolation and gossip [17, 18]. Perceived stigma is the perception of the prevalence of stigmatizing attitudes and behaviours, for example within the general community or in a health facility [19, 20, 21, 22]. Anticipated stigma is the fear that stigma will happen, if for example a family member learns a person is living with HIV, whether or not it is actually experienced [23]. Perceived and anticipated stigma are sometimes described together as felt stigma [24, 25]. Individuals facing stigma may also internalize stigma [22, 25, 26]. Intersecting stigma occurs when individuals who have identities linked to multiple marginalized groups, for example based on socio‐economic status, race, sexual orientation, gender identity or health conditions face multiple intersecting stigmas [27, 28]. Embedded within human rights imperatives, recognition of the necessity and urgency of responding to stigma and resulting discrimination is critical to achieving global targets for HIV testing, linkage to care and viral suppression. As stated in the Global Partnership for Action to Eliminate All Forms of HIV‐Related Stigma and Discrimination [29]:“Without addressing HIV‐related stigma and discrimination, the world will not achieve the goal of ending AIDS as a public health threat by 2030” (p. 5).

Stigma occurs and needs to be addressed in all life spheres and institutions – in the home, community, workplace, places of worship, schools, health care facilities, etc. The health facility offers a logical place to develop and scale‐up a national response to stigma for multiple reasons. It is, in most places, the gateway into HIV treatment and often prevention. As a chronic condition, HIV requires lifelong engagement with the health system. In addition, health care workers are members of their communities and are often looked to for guidance on health and other issues [30, 31, 32]. Health workers are a potentially powerful force not only to reduce stigma in their health facilities, but to be change agents in their homes and communities. Stigma also undermines the health workforce, impacting the health and wellbeing of health workers [33]. The importance of tackling stigma in health facilities is underscored by the UNAIDS‐led global Agenda for Zero Discrimination in Health‐care Settings [34] and corresponding policy guidance [35]. Global initiatives like the Fast Track Cities have also made addressing stigma a key pillar of their response, including a focus on health facilities [36].

The prevalence [37, 38, 39], forms [38, 39, 40, 41, 42, 43] and consequences [4, 7, 8, 9, 12, 13, 33] of health facility HIV stigma are well documented across the globe, as are key drivers of that stigma, including fear of contracting HIV in the workplace, lack of awareness and understanding of stigma, attitudes and the health facility institutional environment [4, 37, 40, 41, 44, 45, 46, 47, 48, 49]. Yet despite this evidence and global recognition of the need to tackle stigma – especially in health facilities – there is little concrete evidence of concerted efforts by countries and donors to scale‐up stigma‐reduction interventions in health facilities within national HIV responses. This may be in part due to the still nascent body of evaluated intervention approaches to stigma‐reduction in health facilities [46, 49, 50, 51, 52, 53, 54, 55, 56].

In Ghana in particular, stigma and discrimination remain a pervasive issue, as documented both through measurement of stigmatizing attitudes in the general population [57] and through the experiences of people living with HIV (PLHIV) [57, 58, 59]. In 2014, only 8.0% of women and 14.1% of men in the general population expressed accepting attitudes as measured by four standardized indicators (caring for a family member in one’s home, buying fresh vegetables from PLHIV, allowing a teacher living with HIV to continue working, and keeping a family member’s positive HIV status secret). PLHIV reported that experienced stigma and discrimination was significant, including social exclusion and gossip, while noting that fear of transmission through casual contact was a key driver of stigma [57]. A health facility stigma assessment in Ghana [58] identified the physical layout of healthcare services; lack of in‐depth knowledge about HIV; fear of contracting the disease; and low levels of respect and dignity based on cultural and moral grounds as contributing factors for stigma and discrimination experienced by PLHIV in health facilities. HIV stigma in Ghana has been documented as a barrier to antiretroviral therapy (ART) services [60]; voluntary testing and counselling (VCT) uptake [61, 62]; and to parents informing children living with HIV of their seropositive status [5, 63].

In its contribution to literature outlining potential interventions within health facility stigma‐reduction, this study evaluates the impact of a “total facility” stigma‐reduction intervention on the drivers and manifestations of stigma and discrimination among health facility staff in Ghana.

## 2 Methods

### 2.1 Setting and Intervention

We evaluated the impact of a total facility stigma‐reduction intervention in five health facilities in Ghana compared to five non‐intervention facilities selected to be broadly comparable with regard to facility size (staff complement and patient load); subnational region; and type of facility (district‐level hospitals). The initial phase of the study collected stigma data from the four highest HIV‐caseload civilian facilities in five regions – Ashanti, Brong Ahafo, Eastern, Greater Accra and Western – for a total of 20 facilities. From these, a subset of 10 facilities were selected for the second phase, with one intervention facility and one comparison facility selected from each region based on staff size (medium‐sized, district‐level facilities) and facility interest to participate. There was no randomization of facilities.

After baseline data collection in June 2017 from health facility staff, we conducted participatory data dissemination and review workshops with staff from all 20 first‐phase facilities in August/September 2017. In these workshops, staff identified stigma and discrimination challenges and then generated potential solutions. Non‐intervention facilities received no further intervention via the study during this period, though some facilities independently conducted stigma‐reduction activities. After the evaluation reported in this manuscript, the non‐intervention facilities are in the process of receiving the intervention approach through scale‐up led by a local Ghanaian organization with support from The Global Fund.

Targeting the whole facility beyond HIV services, the intervention approach included a two‐day participatory stigma‐reduction training for all staff levels (clinical and non‐clinical) with delivery by staff and clients from the facilities who were trained as stigma‐reduction facilitators. Trainings were delivered between September and November 2017 to all categories of staff, with a target of reaching 70% of the facility workforce. The trainings were based on pre‐existing global training materials that are available online [64]. These are targeted towards actionable HIV stigma drivers and were adapted to the Ghanaian context through an in‐country stakeholder workshop. Final Ghanaian training materials are available online [65]. Ultimately, 1228 staff members were trained – 79% of staff at intervention facilities (with individual facilities ranging from 61% to 97% training coverage). Each facility also created an eight to ten member “champion team,” which was provided $5000 USD to develop facility‐specific ancillary activities, including launch events, anti‐stigma‐and‐discrimination banners and posters, additional staff trainings, printed codes of ethics, reporting mechanisms, and staff nametags to enable identification and reporting of stigma and discrimination. More detail on the intervention is available online in a final project report [66]. We conducted follow‐up surveys in April 2018 and disseminated results to staff at the 10 study facilities as well as at a national dissemination meeting for policymakers, donors and community stakeholders.

### 2.2 Sampling

We conducted repeated cross‐sectional surveys, stratified by health facility and job category, of a random sample of staff at the five intervention and five comparison facilities two to three months before and five to six months after the training portion of the intervention. The total sample size was 2308 participants (1154 in each of the pre‐ and post‐intervention periods). To preserve anonymity, we did not attempt to identify the same participants for the follow‐up survey. The sample was designed to provide 80% power to detect a 10‐percentage point change in the only indicator for which pre‐baseline estimates were available: observation of colleagues gossiping about PLHIV. The sampling frame consisted of a complete listing of all staff from which an off‐site statistician generated the target sample. If respondents were unavailable or declined to participate, substitutes were chosen in order from a predetermined list. The survey was designed to be self‐administered if participants could read and to be administered by interviewers to participants who could not read or preferred the administered survey.

### 2.3 Instruments and Variables

Survey instruments are provided in Data S1. The survey included modules to capture demographic information; three actionable stigma and discrimination drivers (perceptions of health facility policies, fears of contracting HIV in the workplace, and attitudes towards PLHIV and key populations); and stigma and discrimination manifestations (self‐reported unnecessary infection control behaviours and observed discrimination by other healthcare workers). The follow‐up survey also included items about exposure to the intervention and perceived changes since baseline.

Survey questions were developed by adapting a globally validated tool for health worker stigma and discrimination [67]. Items were adapted during a one‐day participatory workshop with Ghanaian collaborators and other stakeholders and then tested during a rapid pilot at one health facility which had not been selected for further surveys. Surveys were administered in English, Dangme, Akuapem Twi and Ga. English surveys were translated by professional translators and then checked by mother‐tongue speakers on the research term and any issues resolved with the translators.

The primary outcomes outlined below were composite indicators for each domain, dichotomized from a series of items.

#### 2.3.1 Facility policies

One variable for reporting knowledge that facility guidelines protect PLHIV, men who have sex with men (MSM), and sex workers from discrimination; one variable for believing that the respondent would be punished if discriminating against PLHIV and all of four key populations (MSM, sex workers, sexually active adolescents and persons who inject drugs); and one variable for agreeing or strongly agreeing that the facility has adequate infection control policies and personal protective equipment and knowing that the facility has both a posted post‐exposure prophylaxis (PEP) policy and PEP access.

#### 2.3.2 Fear

Reporting being a little worried, worried, or very worried to conduct one or more of four care activities with clients living with HIV, for fear of HIV acquisition.

#### 2.3.3 Attitudes

Three variables for agreeing or strongly agreeing with one or more stigmatizing statement about PLHIV in general, women living with HIV, and MSM; and one variable for agreeing or strongly agreeing that the respondent would prefer not to provide services to one or more key population.

#### 2.3.4 Self‐reported provision of care in a stigmatizing manner

Reporting usually avoiding physical contact, wearing double gloves, wearing gloves during all aspects of care, or using extra precautions with PLHIV but not other patients.

#### 2.3.5 Observed stigma and discrimination

Observing one or more instances of healthcare workers at the same facility being unwilling to care for, providing poorer care quality to, talking badly about, or disclosing status about PLHIV or one of four key populations in the last six months.

A secondary outcome was participant’s general perception about whether behaviour towards clients had changed in the facility over the year prior to the endline survey, dichotomized as a little/much better versus no change or a little/much worse.

The principal intervention variable was whether the respondent worked at an intervention or comparison facility. Analyses were by intention to treat, so participants were classified as at intervention facilities even if they did not individually receive training, as intention‐to‐treat analysis was expected to capture the effect of average programme implementation. We also collected demographic variables for use in adjusted analyses: age (18 to 24 years, 10 year increments from 24 to 54, and 55+); region; sex; staff category (senior medical, other medical, and administrative or support staff); tenure at the facility (<2 years, two to five years, 5+ years); whether the respondent has ever worked in an HIV‐specific department; and quintiles of the number of PLHIV the respondent reports personally providing care to in the past month.

### 2.4 Statistical analyses

We present basic descriptive statistics of the sample in Table 1. In Table 2, we report the number and proportion of respondents in the follow‐up sample who reported exposure to the main intervention components: trainings in infection control; patients’ rights to informed consent, privacy, and confidentiality; HIV stigma and discrimination; and key population stigma and discrimination; or who participated in any other stigma and discrimination reduction activity since August 2017 when the intervention began.

**Table 1:** Sample characteristics, n (%)

	Comparison, pre‐intervention (N = 555)	Comparison, post‐intervention (N = 555)	Intervention, pre‐intervention (N = 599)	Intervention, post‐intervention (N = 599)	Total (N = 2308)
Age					
18 to 24	56 (10.1%)	22 (4.0%)	80 (13.4%)	41 (6.8%)	199 (8.6%)
25 to 34	263 (47.4%)	230 (41.4%)	253 (42.2%)	272 (45.4%)	1018 (44.1%)
35 to 44	129 (23.2%)	196 (35.3%)	138 (23.0%)	177 (30.0%)	640 (27.7%)
45 to 54	46 (8.3%)	50 (9.0%)	67 (11.2%)	76 (12.7%)	239 (10.4%)
55+	44 (7.9%)	32 (5.8%)	43 (7.2%)	28 (4.7%)	147 (6.4%)
Missing	17 (3.1%)	25 (4.5%)	18 (3.0%)	5 (0.8%)	65 (2.8%)
Sex					
Female	352 (63.4%)	391 (70.5%)	365 (60.9%)	387 (64.6%)	1495 (64.8%)
Male	202 (36.4%)	161 (29.0%)	230 (38.4%)	210 (35.1%)	803 (34.8%)
Missing	1 (0.2%)	3 (0.5%)	4 (0.7%)	2 (0.3%)	10 (0.4%)
Staff category					
Senior medical	26 (4.7%)	35 (6.3%)	39 (6.5%)	47 (7.9%)	147 (6.4%)
Mid‐level medical	410 (73.9%)	435 (78.4%)	458 (76.5%)	450 (75.1%)	1753 (76.0%)
Administrative & support	112 (20.2%)	85 (15.3%)	95 (15.9%)	102 (17.0%)	394 (17.1%)
Missing	7 (1.3%)	0 (0.0%)	7 (1.2%)	0 (0.0%)	14 (0.6%)
Time working at facility					
<2 years	144 (26.0%)	123 (22.2%)	139 (23.2%)	145 (24.2%)	551 (23.9%)
2 to <5 years	170 (30.6%)	164 (30.0%)	160 (26.7%)	192 (32.1%)	686 (29.7%)
5+ years	218 (39.3%)	251 (45.2%)	269 (44.9%)	255 (42.6%)	993 (43.0%)
Missing	23 (4.1%)	17 (3.1%)	31 (5.2%)	7 (1.2%)	78 (3.4%)
Experience working in a clinic specializing in HIV services					
Yes	229 (41.3%)	236 (42.5%)	297 (49.6%)	286 (47.7%)	1048 (45.4%)
No	303 (54.6%)	289 (52.1%)	274 (45.7%)	295 (49.2%)	1161 (50.3%)
Missing	23 (4.1%)	30 (5.4%)	28 (4.7%)	18 (3.0)	99 (4.3%)
Quintiles of number of PLHIV provided care					
Lowest (0 patients)	145 (26.1%)	91 (16.4%)	172 (28.7%)	85 (14.2%)	493 (21.4%)
Second (1 to 3 patients)	85 (15.3%)	73 (13.2%)	60 (10.0%)	65 (10.9%)	283 (12.3%)
Middle (4 to 10 patients)	117 (21.1%)	125 (22.5%)	102 (17.0%)	147 (24.5%)	491 (21.3%)
Fourth (11 to 20 patients)	46 (10.5%)	53 (9.6%)	70 (11.7%)	64 (10.7%)	233 (10.1%)
Highest (21 or more patients)	58 (10.5%)	82 (14.8%)	121 (20.2%)	77 (12.9%)	338 (14.6%)
No response	104 (18.7)	131 (23.6%)	74 (12.4%)	161 (26.9%)	470 (20.4%)
Region					
Ashanti	133 (24.0%)	133 (24.0%)	106 (17.7%)	106 (17.7%)	478 (20.7%)
Brong Ahafo	66 (11.9%)	66 (11.9%)	114 (19.0%)	114 (19.0%)	360 (15.6%)
Eastern	126 (22.7%)	126 (22.7%)	109 (18.2%)	109 (18.2%)	470 (20.4%)
Greater Accra	143 (25.8%)	143 (25.8%)	177 (29.6%)	177 (29.6%)	640 (27.7%)
Western	87 (15.7%)	87 (15.7%)	93 (15.5%)	93 (15.5%)	360 (15.6%)

**Table 2:** Participation in trainings and other activities relevant to stigma and discrimination since August 2017 when the intervention began

Training/activity	Comparison group (N = 555)		Intervention group (N = 599)		*p* value
	n	% (95% CI)	n	% (95% CI)	
Infection control and standard precautions	362	65.2 (53.9 to 75.1)	487	81.3 (74.7 to 86.5)	0.012
Informed consent, privacy, and confidentiality	316	56.9 (41.5 to 71.1)	476	79.5 (73.0 to 84.7)	0.007
HIV stigma and discrimination	249	44.9 (30.7 to 59.9)	507	84.6 (73.5 to 91.6)	0.001
Key population stigma and discrimination	183	33.0 (17.9 to 52.6)	457	76.3 (63.1 to 85.8)	0.002
Any other stigma and discrimination reduction activity	175	31.5 (15.3 to 53.9)	385	64.3 (49.3 to 76.9)	0.020

Confidence interval and the *p*‐value for the difference between the comparison and intervention groups are adjusted for clustering by health facility.

Intervention effects are presented in Table 3 as differences‐in‐differences: the difference in before‐to‐after changes between intervention and comparison areas for each composite indicator. To estimate differences‐in‐differences, we fit saturated linear probability models using generalized linear models with an identity link function and binomial error distribution. Each outcome was regressed on indicator variables for intervention (intervention vs. comparison); time (pre vs. post‐intervention); and their interaction, with the coefficient on the interaction term being the estimated difference‐in‐differences (DID). Primary analyses were conducted on the full sample, and secondary analyses were restricted only to clinical providers. All analyses were by intention‐to‐treat, and we treated facilities designated as comparison but which partially implemented as comparison facilities in the analysis.

In addition to unadjusted estimates, we estimate intervention effects using inverse probability of treatment weighting (IPTW) [68, 69]. The IPTW models generated weights to balance the following covariates across the four intervention‐by‐time groups: subnational region and staff members’ age, sex, employment category, employment tenure, work history in an HIV‐specific department and number of PLHIV personally treated. Separate IPT weights were constructed for the full sample and the clinical staff subsample. We then fit DID models identical to the primary analyses except incorporating IPT weights. We provide full details of the IPTW approach and balance diagnostics in Data S2. All analyses adjust standard errors for clustering by facility.

**Table 3:** Average intervention effects on principal composite outcomes (percentage point difference‐in‐differences)

	Full sample				Medical staff only			
	Unadjusted		IPTW‐adjusted		Unadjusted		IPTW‐adjusted	
	DID (95% CI)	*p* value	DID (95% CI)	*p* value	DID (95% CI)	*p* value	DID (95% CI)	*p* value
Holding 1 + stigmatizing attitude towards								
PLHIV	3.4 (−2.1 to 8.9)	0.225	2.1 (−3.2 to 7.4)	0.437	4.9 (−0.7 to 10.4)	0.086	4.1 (−0.8 to 8.9)	0.101
Women living with HIV	1.7 (−10.2 to 13.7)	0.775	2.1 (−8.9 to 13.1)	0.708	2.8 (−8.8 to 14.5)	0.631	0.8 (−9.9 to 11.6)	0.878
MSM	3.2 (−0.7 to 7.2)	0.111	2.7 (−0.9 to 6.3)	0.142	3.3 (−1.6 to 8.3)	0.184	2.7 (−1.6 to 7.0)	0.212
Preferring not to serve 1 + key population	12.1 (4.2 to 20.0)	0.003	11.1 (3.2 to 19.0)	0.006	15.5 (8.6 to 22.4)	<0.001	12.5 (3.1 to 21.8)	0.009
Fearing to conduct 1 + care activity	–	–	–	–	24.6 (11.7 to 37.5)	<0.001	24.7 (9.6 to 39.8)	0.001
Perception that facility policies protect PLHIV and key populations from discrimination	22.1 (12.6 to 31.6)	<0.001	20.4 (12.7 to 28.0)	<0.001	22.9 (11.6 to 34.2)	<0.001	19.6 (10.3 to 29.0)	<0.001
Believing would get into trouble if discriminate against PLHIV and key populations	8.2 (−1.4 to 17.8)	0.094	11.2 (0.2 to 22.3)	0.046	9.1 (−0.1 to 18.2)	0.052	11.9 (0.8 to 23.0)	0.035
Believing that facility IC policies, PPE, PEP policies, and PEP availability are adequate	18.5 (9.3 to 27.6)	<0.001	17.6 (8.3 to 26.9)	<0.001	18.2 (7.7 to 28.6)	0.001	15.7 (6.5 to 24.8)	0.001
Performing care activities in a stigmatizing/discriminatory way	–	–	–	–	17.8 (4.6 to 31.0)	0.008	20.0 (4.0 to 36.1)	0.015
Observing incidents of stigmatizing/discriminatory care by other staff	9.6 (3.4 to 15.9)	0.003	7.4 (−0.3 to 15.2)	0.060	12.5 (5.2 to 19.8)	0.001	11.3 (2.8 to 19.7)	0.009

	Difference (95% CI)	*p* value	Difference (95% CI)	*p* value	Difference (95% CI)	*p* value	Difference (95% CI)	*p* value
Believing conduct toward PLHIV has improved since one year ago	17.9 (10.1 to 25.7)	0.001	19.0 (12.2 to 25.8)	0.001	18.3 (10.0 to 26.6)	0.002	18.0 (11.2 to 24.9)	0.001

All estimates are constructed such that a positive DID is an improvement (even if the original measure was scaled so that higher numbers were an undesirable outcome). Fear and avoidance outcomes are restricted only to clinical staff because the sizable majority of non‐clinical staff answered “not applicable” to the single item applicable to them. DID, difference‐in‐differences; IPTW, inverse probability of treatment weighting; MSM, men who have sex with men; PEP, post‐exposure prophylaxis.

In the main analysis, we did not adjust for multiple comparisons because all outcomes were theoretically informed and predetermined and no analyses were added *post hoc*. Because some readers might expect adjustment; however, we present an analysis in Data S4 that reports risk of false discovery because of multiple outcomes. We present false discovery rates as q‐values produced via Simes’ method. We used Stata v15.1 for all analyses and statistical code and output are provided in Data S3.

The institutional review boards at Health Media Labs and the Ghana Health Services Ethical Review Board approved the study, and all participants provided written informed consent.

## 3 Results

Characteristics of the analytical sample of 2308 participants (1154 in each of the pre‐ and post‐intervention periods) are in Table 1. They are broadly comparable across the four intervention‐by‐time groups, and balance of characteristics before and after IPT weighting is provided in Data S2. IPT‐weighted models met generally accepted criteria for balance of potential confounders [70].

As expected, exposure to trainings and other activities to reduce stigma and discrimination was higher among intervention than comparison staff during the post‐intervention period, with each of the trainings reaching at least three‐quarters of intervention respondents, and about two‐thirds being exposed to at least one additional intervention activity (Table 2). More than half of comparison respondents reported receiving training on infection control and universal precautions (65%) and informed consent, privacy, and confidentiality (57%) during the same time period. A lower but still substantial percentage of comparison respondents reported receiving HIV stigma and discrimination training (45%), key population stigma and discrimination training (33%) and other relevant activities (32%).

Using intention‐to‐treat analyses, we observed favourable intervention effects for all outcome domains except for stigmatizing attitudes in both the full sample and the medical staff‐only subsample (Table 3). In the IPTW‐adjusted analyses of the full sample, preferring not to provide services to PLHIV or a key population member improved by 11.1 percentage points (95% CI 3.2 to 19.0) more in intervention than comparison facility respondents. Knowledge of policies to protect against discrimination (DID = 20.4 percentage points [pp], 95% CI 12.7 to 28.0); belief that discrimination would be punished (DID = 11.2pp, 95% CI 0.2 to 22.3); and knowledge of and belief in the adequacy of infection control policies (DID = 17.6pp, 95% CI 8.3 to 26.9) all improved significantly. Observing stigma and discrimination incidents improved by 7.4 percentage points more among intervention than comparison facility respondents (95% CI − 0.3 to 15.2), though this result was only marginally significant (*p* = 0.06). Results were very similar in the unadjusted analyses.

We found comparable improvements in the subsample composed only of medical staff (Table 3). Here too, there were no significant improvements in stigmatizing attitudes, but all other outcome domains improved significantly with similar intervention effect estimates to those identified in the full sample. In addition to the domains measured in the full sample, two domains were limited only to medical staff. Fear of conducting at least one care activity improved by 24.7 more percentage points (95% CI 9.6 to 39.8) among intervention than comparison respondents in the IPTW‐adjusted model, and using unnecessary infection control precautions when caring for PLHIV but not other patients improved by 20.0 more percentage points (95% CI 4.0 to 36.1) among intervention than comparison respondents. Unadjusted results were comparable.

When asked whether there had been a change in behaviour towards PLHIV over the last year, intervention facility respondents were 19.0 percentage points (95% CI 12.2 to 25.8) more likely to say it was better in IPTW‐adjusted analyses (Table 3). Results were similar in the medical staff subpopulation (18.0pp; 95% CI 11.2 to 24.9) and in unadjusted analyses. Substitution of false discovery rate q‐values for *p*‐values does not meaningfully change conclusions.

## 4 Discussion

Evidence continues to grow of the negative relationship of stigma to pre‐exposure prophylaxis [1], HIV testing [2, 3, 4], linkage and retention in care [5, 6, 7], medication adherence [8, 9, 10, 11, 12, 13] and viral load suppression [2, 14, 15]. More visible global recognition of stigma in fuelling the HIV epidemic and in undermining the HIV response has been forthcoming through recent global declarations [29, 35, 71]. This includes a recognition of the importance of addressing stigma in health facilities [72], yet concerted efforts to tackle stigma at any scale within countries are lacking, apart from Thailand [73]. One reason for such inaction may be the small, but growing, body of evidence of effective intervention models for HIV‐stigma reduction, particularly in health facilities [46, 49, 50, 51, 52, 53, 54, 55, 56]. This paper adds to that literature.

This evaluation of the Ghana “total facility” intervention found strong and consistent evidence of improvements, across indicator domains, apart from stigmatizing attitudes. Fear of acquiring HIV while providing care for clients living with HIV improved significantly in intervention versus comparison facilities, as did associated stigmatizing avoidance behaviours such as double gloving. These findings mirror results of interventions in China [55] and Vietnam [56] that also included addressing fear of HIV transmission in the health facility as a key component of stigma‐reduction interventions. A further study in China underscores the importance of addressing fear directly and through a focus on standard precautions as a key strategy in reducing health facility stigma and discrimination [74]. There was no statistically significant DID between the intervention and control facilities on the composite stigmatizing attitudes variable. This is not unexpected as changing deeply held attitudes may require a longer and more intense intervention than changing fear and behaviours based in incorrect knowledge or strengthening the facility environment to facilitate delivery of non‐stigmatizing care. Additionally, more items in the composite attitude variables results in less change being expected because all of a respondent’s stigmatizing attitudes would have to be reduced to alter a single “none‐or‐any” composite. That changing (and measuring change in) stigmatizing attitudes is potentially more complicated than for other immediately actionable drivers of stigma is also highlighted by a study in Vietnam, where the arm of the study that received a focus on social stigma (attitudes) had a similar drop in stigmatizing attitudes to the arm that received only a focus on fear‐based stigma [56].

There are limitations to this study that should be noted. The short‐time frame of the evaluation did not allow for assessment of longer‐term intervention effects and therefore, could overestimate more transient effects of the intervention or underestimate those that require longer intervention periods. The nature of the setting, design and funding did not allow for randomization of the facilities to intervention or comparison. However, facility selection, the DID approach and IPTW should substantially account for differences between the intervention and comparison groups. Some stigma‐reduction activities did occur in the comparison group of facilities, which likely diminishes some of the intervention effect. All facilities received their baseline results before the intervention began in the intervention sites, as required by ethical guidelines, which may have spurred some comparison facilities to begin addressing their baseline results. As presented in Table 2, respondents in the comparison arm did report participating in a range of training related to specific stigma drivers (e.g. Infection control and standard precautions) and other general, non‐specified stigma‐reduction activities. While the percent of staff reporting such exposure was significantly lower in comparison to intervention sites, the data does indicate some activities were happening in the comparison facilities. Social desirability bias is always present in stigma‐reduction studies. One way that the study worked to reduce this potential bias was by implementing data collection strategies to reassure health care staff respondents that their responses were anonymous, including self‐filled questionnaires (where literacy was not a challenge); placing of questionnaires by respondents in envelopes which they sealed and that were put in a locked box; and not collecting any personal identifiers on the questionnaire. That the attitudes indicator, one that we would expect to be particularly sensitive to social desirability bias, was the one indicator that did not change significantly perhaps indicates a mitigation of social desirability in the study.

The rationale for addressing stigma for an effective HIV response and to achieve national and global targets is clearly underscored by the evidence of the negative link between stigma and testing [2, 3], treatment [10, 11, 12, 13] and viral load suppression [2, 14, 15]. The importance and urgency of responding to stigma in a consistent and scaled manner is visible in recent global calls for stigma‐reduction initiatives [29, 35, 71, 72]. Translating these global calls into action on the ground will require national HIV responses to include stigma‐reduction as a core feature, alongside prevention and treatment. Health facilities are a logical and feasible place to initiate this work, as demonstrated by this study and others. Ghana has begun building on this pilot intervention, expanding the intervention to other facilities with The Global Fund’s support, while Thailand is in the midst of expanding approaches beyond a pilot study, with their stigma‐reduction intervention currently implemented in over 100 health facilities. (Personal Communication, Dr. Taweesap Siraprapasiri, Thailand MOPH).

## 5 Conclusion

Addressing HIV stigma is not only a public health imperative but a human rights one. Tackling HIV stigma in the health system is a logical place to develop and scaleup a national response to HIV stigma. This paper adds to the small but growing literature demonstrating that HIV stigma can effectively be addressed in health facilities and is a challenge welcomed by facility staff and management. This evidence provides a solid foundation for developing and testing the feasibility and efficacy of scaling up health facility stigma‐reduction within countries. It will be critical that these efforts are accompanied by rigorous implementation science to ensure ongoing learning and adaptation to maximize effectiveness and long‐term impact.

## Competing interest

The authors declare that they have no competing interests.

## Authors’ contributions

LN, NAA, KA, NA, EG, SJ, PM, CS and RV conceived, designed, and implemented the study. JDK and RV analysed the data. LN, ML, PM and JDK drafted portions of the manuscript. All authors provided data interpretation and revised the manuscript critically for intellectual content. All authors approved the final version of the manuscript.

## Supporting information


**Data S1.** Survey questionnaires.

**Data S2.** Inverse probability of treatment weighting models.

**Data S3. **Replication log for Ghana facility intervention Diff‐in‐Diff. 21 August 2019**.

**Table S4. **False discovery rate q‐values that account for assessing multiple, correlated outcomes
