# Supplementary material for: Results from a difference‐in‐differences evaluation of health facility HIV and key population stigma‐reduction interventions in Ghana
Source: J Int AIDS Soc. 2020 Apr 23;23(4):e25483. doi: 10.1002/jia2.25483 (PMC7180216; doi:10.1002/jia2.25483)
Supplement: Supplementary file 1 — Data S1. Survey questionnaires. [file JIA2-23-e25483-s001.pdf]

Date of data Collection:

Name of Interviewer:

Name of Facility:

Facility Code:

Participation Identification Code (PID) Number:

Field Supervisor:

UNDERSTANDING HIV RELATED STIGMA AND DISCRIMINATION AMONG HEALTH FACILITY STAFF (INTERVENTION)

ENGLISH

SECTION 1: BACKGROUND INFORMATION

First we would like to ask about your background.

1. Please state your age from the following ranges: ☐ 18-24 ☐ 25-34 ☐ 35-44 ☐ 45-54 ☐ 55 and over

2. What is your sex?

   ☐ Female ☐ Male

3. What is the highest level of school you completed?

   ☐ None ☐ Primary/Basic/Middle School ☐ Technical/Vocational ☐ Secondary ☐ Tertiary

   ☐ Other: [ ]

4. What is your current job in this facility **(the job you spend the majority of your time on)**?

   ☐ Accountant/Cashier/Revenue Collector ☐ Cleaning Staff/Orderly

   ☐ Dentist ☐ Dental Technician/Hygienist ☐ Doctor

   ☐ Nurse Assistant ☐ Nurse ☐Midwife ☐ Receptionist

   ☐ Physician Assistant ☐ Medical Records Personnel ☐ Pharmacist

   ☐ Pharmacist Technologist ☐ Biomedical Scientist/Laboratory Technician

   ☐ Peer Educator /Model of Hope ☐ Security Staff

   ☐ Other: [ ]

5. How many years have you been working at this facility? (If less than 1 year, only fill in the number of months)

   [ ] Years [ ] Months

6. Have you ever worked in a department that specializes in offering services in HIV care and treatment?

   ☐Yes ☐ No

7. ***In the past one month***, approximately how many HIV-positive people have you personally provided with care or services? [ ][ ][ ]

8. Have you received any form of training in the following subjects since August 2017? (Check all that apply.)

	Yes	No
a. Infection control and universal precautions (including post-exposure prophylaxis - PEP)	☐	☐

b. Patients' informed consent, privacy, and confidentiality ☐ ☐

c. HIV stigma and discrimination ☐ ☐

d. Key population stigma and discrimination ☐ ☐

e. Have you participated in any other stigma and discrimination reduction activities in your facility? ☐ ☐

SECTION 2: INFECTION CONTROL

Now we will ask you about HIV infection concerns in your health facility.

9. How worried would you be about getting HIV if you did the following?
If any of the following is not one of your job responsibilities, please select "Not applicable."

i. Touched the clothing or bedding of a person living with HIV
☐ Not worried ☐ A little worried ☐ Worried ☐ Very worried ☐ Not applicable

ii. Dressed the wounds of a person living with HIV
☐ Not worried ☐ A little worried ☐ Worried ☐ Very worried ☐ Not applicable

iii. Drew blood from a person living with HIV
☐ Not worried ☐ A little worried ☐ Worried ☐ Very worried ☐ Not applicable

iv. Took the temperature, blood pressure or pulse of a patient living with HIV
☐ Not worried ☐ A little worried ☐ Worried ☐ Very worried ☐ Not applicable

10. Do you usually use any of the following measures when providing care or services for a person living with HIV? ***If any of the following are not applicable to your job responsibilities, please select "Not applicable."***

a. Avoid physical contact
☐ Yes ☐ No ☐ Not applicable

b. Wear double gloves
☐ Yes ☐ No ☐ Not applicable

c. Wear gloves during all aspects of the patient's care
☐ Yes ☐ No ☐ Not applicable

d. Use any extra precautionary measures with persons living with HIV that you do not use with other patients
☐ Yes ☐ No ☐ Not applicable

SECTION 3: HEALTH FACILITY ENVIRONMENT

Now we will ask about practices in your health facility and your experiences working in a facility that provides care to people living with HIV and other key populations.

11. In the past 6 months, how often have you observed healthcare workers unwilling to care for:

a. Persons living with or thought to be living with HIV?
☐ Never ☐ Once or twice ☐ Several times ☐ Most of the time

b. Men who have sex with men or thought to be having sex with men?
☐ Never ☐ Once or twice ☐ Several times ☐ Most of the time

c. A sex worker or people thought to be sex workers?
☐ Never ☐ Once or twice ☐ Several times ☐ Most of the time

d. A sexually active adolescent/youth or thought to be sexually active adolescent/youth?
☐ Never ☐ Once or twice ☐ Several times ☐ Most of the time

e. A person who injects drugs for non-medical reasons or thought to be injecting drugs?
☐ Never ☐ Once or twice ☐ Several times ☐ Most of the time

12. In the past 6 months, how often have you observed healthcare workers providing poorer quality of care for:

a. Persons living with or thought to be living with HIV?
☐ Never ☐ Once or twice ☐ Several times ☐ Most of the time

b. Men who have sex with men or thought to be having sex with men?
☐ Never ☐ Once or twice ☐ Several times ☐ Most of the time

c. A sex worker or people thought to be sex workers?
☐ Never ☐ Once or twice ☐ Several times ☐ Most of the time

d. A sexually active adolescent/youth or thought to be a sexually active adolescent/youth?
☐ Never ☐ Once or twice ☐ Several times ☐ Most of the time

e. A person who injects drugs for non-medical reasons or thought to be injecting drugs?
☐ Never ☐ Once or twice ☐ Several times ☐ Most of the time

13. In the past 6 months, how often have you observed healthcare workers talking badly about:

a. Persons living with or thought to be living with HIV?
☐ Never ☐ Once or twice ☐ Several times ☐ Most of the time

b. Men who have sex with men or thought to be having sex with men?
☐ Never ☐ Once or twice ☐ Several times ☐ Most of the time

c. A sex worker or people thought to be sex workers?
☐ Never ☐ Once or twice ☐ Several times ☐ Most of the time

d. A sexually active adolescent/youth or thought to be sexually active adolescent/youth?
☐ Never ☐ Once or twice ☐ Several times ☐ Most of the time

e. A person who injects drugs for non-medical reasons or thought to be injecting drugs for non-medical reasons?
☐ Never ☐ Once or twice ☐ Several times ☐ Most of the time

14. In the past 6 months, how often have you observed healthcare workers disclosing a person living with HIV or other status without their consent, in a situation that was not medically required?
☐ Never ☐ Once or twice ☐ Several times ☐ Most of the time

15. How worried are you about:

a. Friends and family treating you differently because you care for persons living with HIV?
☐Never worried ☐Worried ☐Sometimes worried ☐Always worried ☐Not applicable

b. Friends and family treating you differently because you care for persons who are men who have sex with men?
☐Never worried ☐Worried ☐Sometimes worried ☐Always worried ☐Not applicable

16. In your opinion, how hesitant are healthcare workers to take an HIV test, in this facility, due to fear of other people's reactions if the test is positive?

☐ Not hesitant ☐ A little hesitant ☐ Somewhat hesitant ☐ Very hesitant

17. How hesitant are you to take an HIV test, in this facility, due to fear of other people's reactions if the test is positive?

☐ Not hesitant ☐ A little hesitant ☐ Somewhat hesitant ☐ Very hesitant

18. In your opinion, how hesitant are healthcare workers in this facility to work alongside a co-worker living with HIV, regardless of their duties?
☐ Not hesitant ☐ A little hesitant ☐ Somewhat hesitant ☐ Very hesitant

19. How confident are you that when you take an HIV test in this facility the results will be confidential?
☐ Not confident ☐ A little confident ☐ Somewhat confident ☐ Very Confident

20. In your opinion, how hesitant do you think a healthcare worker living with HIV would be to seek healthcare in this facility?

☐ Not hesitant ☐ A little hesitant ☐ Somewhat hesitant ☐ Very hesitant

21. In your opinion, how hesitant are your co-worker(s) to care for :

a. People living with or thought to be living with HIV ?

☐ Not hesitant ☐ A little hesitant ☐ Somewhat hesitant ☐ Very hesitant

b. Men who have sex with men or thought to be having sex with men?

☐ Not hesitant ☐ A little hesitant ☐ Somewhat hesitant ☐ Very hesitant

c. Sex workers or people thought to be sex workers?

☐ Not hesitant ☐ A little hesitant ☐ Somewhat hesitant ☐ Very hesitant

d. Sexually active adolescents/youth or thought to be sexually active adolescents/youth?

☐ Not hesitant ☐ A little hesitant ☐ Somewhat hesitant ☐ Very hesitant

e. People who inject drugs for non-medical reasons or thought to inject drugs?

☐ Not hesitant ☐ A little hesitant ☐ Somewhat hesitant ☐ Very hesitant

SECTION 4: HEALTH FACILITY POLICIES

Now we are going to ask about the institutional policy and work environment in your facility.

22. In my facility nobody allowed to test a patient for HIV without his/her knowledge.
☐ Yes ☐ No ☐ Don't Know

23. I will get into trouble at work if I discriminate against persons living with HIV.
☐ Yes ☐ No ☐ Don't Know

24. I will get into trouble at work if I discriminate against patients who are men who have sex with men.
☐ Yes ☐ No ☐ Don't Know

25. I will get into trouble at work if I discriminate against patients who are sex workers
☐ Yes ☐ No ☐ Don't Know

26. I will get into trouble at work if I discriminate against a sexually active adolescent/youth.
☐ Yes ☐ No ☐ Don't Know

27. I will get into trouble at work if I discriminate against patients who inject drugs for non-medical reasons.

☐ Yes ☐ No ☐ Don't Know

28. Do you strongly agree, agree, disagree, or strongly disagree with the following statements?

a. There are adequate supplies (personal protective equipment (PPE)) in my health facility that reduce my risk of becoming infected with HIV.
☐ Strongly Agree ☐ Agree ☐ Disagree ☐ Strongly Disagree

b. There are standardized procedures/protocols in my health facility that reduce my risk of becoming infected with HIV.

☐ Strongly Agree ☐ Agree ☐ Disagree ☐ Strongly Disagree

29. My health facility has written guidelines to protect persons living with HIV from discrimination.
☐ Yes ☐ No ☐ Don't Know

30. My health facility has written guidelines to protect patients who are men who have sex with men from discrimination.
☐ Yes ☐ No ☐ Don't Know

31. My health facility has written guidelines to protect patients who are sex workers from discrimination.
☐ Yes ☐ No ☐ Don't Know

32. Does your facility have post-exposure prophylactic (PEP) protocol placed where all staff can see?
☐ Yes ☐ No

33. Do you have access to post-exposure prophylactic (PEP) medications in your health facility?

☐ Yes ☐ No ☐ Don't Know

SECTION 5: OPINIONS ABOUT PEOPLE LIVING WITH HIV AND OTHER KEY POPULATIONS.

Now we are going to ask about opinions related to people living with HIV and other key populations.

34. Do you strongly agree, agree, disagree, or strongly disagree with the following statements?

a. Most people living with HIV do not care if they infect other people.
☐ Strongly Agree ☐ Agree ☐ Disagree ☐ Strongly Disagree

b. People living with HIV should feel ashamed of themselves.
☐ Strongly Agree ☐ Agree ☐ Disagree ☐ Strongly Disagree

c. Most people living with HIV have had many sexual partners.
☐ Strongly Agree ☐ Agree ☐ Disagree ☐ Strongly Disagree

d. Most female sex workers are living with HIV
☐ Strongly Agree ☐ Agree ☐ Disagree ☐ Strongly Disagree

e. People get infected with HIV because they engage in irresponsible behaviors.
☐ Strongly Agree ☐ Agree ☐ Disagree ☐ Strongly Disagree

f. HIV is punishment for bad behavior
☐ Strongly Agree ☐ Agree ☐ Disagree ☐ Strongly Disagree

g. Most men living with HIV are men who have sex with other men
☐ Strongly Agree ☐ Agree ☐ Disagree ☐ Strongly Disagree

h. Men who have sex with men were born that way.
☐ Strongly Agree ☐ Agree ☐ Disagree ☐ Strongly Disagree

i. Women living with HIV should be allowed to have babies if they wish.
☐ Strongly Agree ☐ Agree ☐ Disagree ☐ Strongly Disagree

j. I would be ashamed if I had HIV.
☐ Strongly Agree ☐ Agree ☐ Disagree ☐ Strongly Disagree

k. I would be ashamed if someone in my family had HIV.
☐ Strongly Agree ☐ Agree ☐ Disagree ☐ Strongly Disagree

l. A woman living with HIV should be provided treatment only if she is using family planning methods.
☐ Strongly Agree ☐ Agree ☐ Disagree ☐ Strongly Disagree

35. Do you strongly agree, agree, disagree, or strongly disagree with the following statements?

a. If I had a choice, I would prefer not to provide services to men who have sex with men.
- ☐ Strongly Agree
- ☐ Agree
- ☐ Disagree
- ☐ Strongly Disagree

b. If I had a choice, I would prefer not to provide services to sex workers
- ☐ Strongly Agree
- ☐ Agree
- ☐ Disagree
- ☐ Strongly Disagree

c. If I had a choice, I would prefer not to provide HIV and other sexual and reproductive health (SRH) services to sexually active adolescents.
- ☐ Strongly Agree
- ☐ Agree
- ☐ Disagree
- ☐ Strongly Disagree

d. It should be optional for healthcare workers to care for key populations (MSM, sex workers and drug users).
- ☐ Strongly Agree
- ☐ Agree
- ☐ Disagree
- ☐ Strongly Disagree

SECTION 6: FEEDBACK FROM INTERVENTION

<table>
<tr><td colspan="2">36. Have you participated in the HIV stigma and discrimination reduction training organized since August 2017 in this facility?

☐ Yes ☐ No ☐ Don’t know</td></tr>
<tr><td>(If <u>yes</u>, please answer Question a below)</td><td>(If <u>no</u> or <u>don’t know</u>, please answer Questions b and c below)</td></tr>
<tr><td rowspan="2">a. How many days did you participate in the training?

☐ Half Day or Less ☐ One Day ☐ One and Half Days ☐ Two Days</td><td>b. Did you hear about the HIV stigma and discrimination reduction training?

☐ Yes ☐ No</td></tr>
<tr><td>c. Would you like to participate in the HIV stigma reduction training at this facility?

☐ Yes ☐ No</td></tr>
</table>

37. Have you heard of the new HIV stigma and discrimination reduction initiative in this facility?

☐ Yes ☐ No

a. If yes, did you participate in the initiative launch?

☐ Yes ☐ No

38. Have you received any of the following new HIV stigma and discrimination reduction materials at this facility since August 2017?

1. Quantity, Quality and Route of transmission materials (QQR)

   ☐ Yes ☐ No

2. Code of ethics

   ☐ Yes ☐ No

3. Standard precautions guidelines

   ☐ Yes ☐ No

39. Have you seen the stigma and discrimination depicting posters at this facility since August 2017?

☐ Yes ☐ No

40. Have you had any organized discussions about stigma and discrimination during your departmental staff meetings or durbars since August 2017?

☐ Yes ☐ No

41. Please describe any other stigma and discrimination-reduction activity in which you participated at this facility.

____

____

____

42. Do you know about the stigma and discrimination reduction champion team in this facility?

☐ Yes, I am a member

☐ Yes, I have interacted with a team member

☐ No

43. Compared to this time last year, do you think there has been a change in behavior towards clients living with HIV in this facility?

☐ Much worse ☐ A little worse ☐ No change ☐ A little better ☐ Much better

44. Have you taken any action to change or challenge stigma and discrimination in this facility since August 2017, as a result of any of the stigma and discrimination reduction activities?

☐ Yes ☐ No

If yes, please describe:

____

____

____

45. Do you provide any services (medical or non-medical) to pregnant women or do any work in the following departments: antenatal care, prevention of mother to child transmission, labor or delivery wards, or postpartum care?

☐ Yes **CONTINUE ON TO SECTION 7, Q46 (next page).**

☐ No **YOU HAVE COMPLETED THE QUESTIONNAIRE. THANK YOU FOR SHARING**

YOUR VALUABLE TIME AND INSIGHTS WITH US.

SECTION 7: ANTENATAL CARE, PREVENTION OF MOTHER-TO-CHILD TRANSMISSION, LABOR AND DELIVERY WARDS

The following section is to be completed by any staff who works in the following departments: antenatal care, prevention of mother-to-child transmission of HIV, labor and delivery rooms and post-natal care.

46. How worried are you about assisting in labor and delivery

a. If the woman is living with HIV?
☐ Not worried ☐ A little worried ☐ Worried ☐ Very worried ☐ Not applicable

b. If the woman had been on anti-retroviral (ARV) treatment for six months or more
☐ Not worried ☐ A little worried ☐ Worried ☐ Very worried ☐ Not applicable

c. If the woman has been on anti-retroviral (ARV) treatment for less than a month or not at all
☐ Not worried ☐ A little worried ☐ Worried ☐ Very worried ☐ Not applicable

47. ***In the past 6 months***, how often have you observed other healthcare providers:

a) Performing an HIV test on a pregnant woman without her informed consent during ANC?
☐ Never ☐ Once or twice ☐ Several times ☐ Most of the time

b) Performing an HIV test on a pregnant woman without her informed consent during labor and delivery?
☐ Never ☐ Once or twice ☐ Several times ☐ Most of the time

c) Neglecting a woman living with HIV during labor and delivery because of her HIV status?
☐ Never ☐ Once or twice ☐ Several times ☐ Most of the time

d) Using additional infection-control procedures (e.g., double gloves) with a pregnant woman living with HIV during labor and delivery because of her HIV status?
☐ Never ☐ Once or twice ☐ Several times ☐ Most of the time

e) Disclosing the status of a pregnant woman living with HIV to others without her consent?
☐ Never ☐ Once or twice ☐ Several times ☐ Most of the time

48. Do you strongly agree, agree, disagree, or strongly disagree with the following statements?

a) If a pregnant woman is HIV positive, her partner/child's father has a right to know.
☐ Strongly Agree ☐ Agree ☐ Disagree ☐ Strongly Disagree

b) Pregnant women who refuse HIV testing are irresponsible.
☐ Strongly Agree ☐ Agree ☐ Disagree ☐ Strongly Disagree

c) Women living with HIV should not get pregnant if they already have children.
☐ Strongly Agree ☐ Agree ☐ Disagree ☐ Strongly Disagree

d) It can be appropriate to sterilize a woman living with HIV, even if this is not her choice.
☐ Strongly Agree ☐ Agree ☐ Disagree ☐ Strongly Disagree

e) Women living with HIV should not breastfeed their babies
☐ Strongly Agree ☐ Agree ☐ Disagree ☐ Strongly Disagree

Thank You

Date of data Collection:

Name of Interviewer:

Name of Facility:

Facility Code:

Participation Identification Code (PID) Number:

Field Supervisor:

UNDERSTANDING HIV RELATED STIGMA AND DISCRIMINATION AMONG HEALTH FACILITY STAFF (COMPARISON)

ENGLISH

SECTION 1: BACKGROUND INFORMATION

First we would like to ask about your background.

1. Please state your age from the following ranges: ☐ 18-24 ☐ 25-34 ☐ 35-44 ☐ 45-54 ☐ 55 and over

2. What is your sex?

   ☐ Female ☐ Male

3. What is the highest level of school you completed?

   ☐ None ☐ Primary/Basic/Middle School ☐ Technical/Vocational ☐ Secondary ☐ Tertiary

   ☐ Other: [ ]

4. What is your current job in this facility **(the job you spend the majority of your time on)**?

   ☐ Accountant/Cashier/Revenue Collector ☐ Cleaning Staff/Orderly

   ☐ Dentist ☐ Dental Technician/Hygienist ☐ Doctor

   ☐ Nurse Assistant ☐ Nurse ☐Midwife ☐ Receptionist

   ☐ Physician Assistant ☐ Medical Records Personnel ☐ Pharmacist

   ☐ Pharmacist Technologist ☐ Biomedical Scientist/Laboratory Technician

   ☐ Peer Educator /Model of Hope ☐ Security Staff

   ☐ Other: [ ]

5. How many years have you been working at this facility? (If less than 1 year, only fill in the number of months)

   [ ] Years [ ] Months

6. Have you ever worked in a department that specializes in offering services in HIV care and treatment?
   ☐Yes ☐ No

7. ***In the past one month***, approximately how many HIV-positive people have you personally provided with care or services? [ ][ ][ ]

8. Have you received any form of training in the following subjects since August 2017? (Check all that apply.)

	Yes	No
a. Infection control and universal precautions (including post-exposure prophylaxis - PEP)	☐	☐

b. Patients' informed consent, privacy, and confidentiality ☐ ☐

c. HIV stigma and discrimination ☐ ☐

d. Key population stigma and discrimination ☐ ☐

e. Have you participated in any other stigma and discrimination reduction activities in your facility? ☐ ☐

SECTION 2: INFECTION CONTROL

Now we will ask you about HIV infection concerns in your health facility.

9. How worried would you be about getting HIV if you did the following?

If any of the following is not one of your job responsibilities, please select "Not applicable."

i. Touched the clothing or bedding of a person living with HIV
☐ Not worried ☐ A little worried ☐ Worried ☐ Very worried ☐ Not applicable

ii. Dressed the wounds of a person living with HIV
☐ Not worried ☐ A little worried ☐ Worried ☐ Very worried ☐ Not applicable

iii. Drew blood from a person living with HIV
☐ Not worried ☐ A little worried ☐ Worried ☐ Very worried ☐ Not applicable

iv. Took the temperature, blood pressure or pulse of a patient living with HIV
☐ Not worried ☐ A little worried ☐ Worried ☐ Very worried ☐ Not applicable

10. Do you usually use any of the following measures when providing care or services for a person living with HIV? ***If any of the following are not applicable to your job responsibilities, please select "Not applicable."***

a. Avoid physical contact
☐ Yes ☐ No ☐ Not applicable

b. Wear double gloves
☐ Yes ☐ No ☐ Not applicable

c. Wear gloves during all aspects of the patient's care
☐ Yes ☐ No ☐ Not applicable

d. Use any extra precautionary measures with persons living with HIV that you do not use with other patients
☐ Yes ☐ No ☐ Not applicable

SECTION 3: HEALTH FACILITY ENVIRONMENT

Now we will ask about practices in your health facility and your experiences working in a facility that provides care to people living with HIV and other key populations.

11. In the past 6 months, how often have you observed healthcare workers unwilling to care for:

a. Persons living with or thought to be living with HIV?
☐ Never ☐ Once or twice ☐ Several times ☐ Most of the time

b. Men who have sex with men or thought to be having sex with men?
☐ Never ☐ Once or twice ☐ Several times ☐ Most of the time

c. A sex worker or people thought to be sex workers?
☐ Never ☐ Once or twice ☐ Several times ☐ Most of the time

d. A sexually active adolescent/youth or thought to be sexually active adolescent/youth?
☐ Never ☐ Once or twice ☐ Several times ☐ Most of the time

e. A person who injects drugs for non-medical reasons or thought to be injecting drugs?
☐ Never ☐ Once or twice ☐ Several times ☐ Most of the time

12. In the past 6 months, how often have you observed healthcare workers providing poorer quality of care for:

a. Persons living with or thought to be living with HIV?
☐ Never ☐ Once or twice ☐ Several times ☐ Most of the time

b. Men who have sex with men or thought to be having sex with men?
☐ Never ☐ Once or twice ☐ Several times ☐ Most of the time

c. A sex worker or people thought to be sex workers?
☐ Never ☐ Once or twice ☐ Several times ☐ Most of the time

d. A sexually active adolescent/youth or thought to be a sexually active adolescent/youth?
☐ Never ☐ Once or twice ☐ Several times ☐ Most of the time

e. A person who injects drugs for non-medical reasons or thought to be injecting drugs?
☐ Never ☐ Once or twice ☐ Several times ☐ Most of the time

13. In the past 6 months, how often have you observed healthcare workers talking badly about:

a. Persons living with or thought to be living with HIV?
☐ Never ☐ Once or twice ☐ Several times ☐ Most of the time

b. Men who have sex with men or thought to be having sex with men?
☐ Never ☐ Once or twice ☐ Several times ☐ Most of the time

c. A sex worker or people thought to be sex workers?
☐ Never ☐ Once or twice ☐ Several times ☐ Most of the time

d. A sexually active adolescent/youth or thought to be sexually active adolescent/youth?
☐ Never ☐ Once or twice ☐ Several times ☐ Most of the time

e. A person who injects drugs for non-medical reasons or thought to be injecting drugs for non-medical reasons?
☐ Never ☐ Once or twice ☐ Several times ☐ Most of the time

14. In the past 6 months, how often have you observed healthcare workers disclosing a person living with HIV or other status without their consent, in a situation that was not medically required?
☐ Never ☐ Once or twice ☐ Several times ☐ Most of the time

15. How worried are you about:

a. Friends and family treating you differently because you care for persons living with HIV?
☐Never worried ☐Worried ☐Sometimes worried ☐Always worried ☐Not applicable

b. Friends and family treating you differently because you care for persons who are men who have sex with men?
☐Never worried ☐Worried ☐Sometimes worried ☐Always worried ☐Not applicable

16. In your opinion, how hesitant are healthcare workers to take an HIV test, in this facility, due to fear of other people's reactions if the test is positive?

☐ Not hesitant ☐ A little hesitant ☐ Somewhat hesitant ☐ Very hesitant

17. How hesitant are you to take an HIV test, in this facility, due to fear of other people's reactions if the test is positive?

☐ Not hesitant ☐ A little hesitant ☐ Somewhat hesitant ☐ Very hesitant

18. In your opinion, how hesitant are healthcare workers in this facility to work alongside a co-worker living with HIV, regardless of their duties?

☐ Not hesitant ☐ A little hesitant ☐ Somewhat hesitant ☐ Very hesitant

19. How confident are you that when you take an HIV test in this facility the results will be confidential?

☐ Not confident ☐ A little confident ☐ Somewhat confident ☐ Very Confident

20. In your opinion, how hesitant do you think a healthcare worker living with HIV would be to seek healthcare in this facility?

☐ Not hesitant ☐ A little hesitant ☐ Somewhat hesitant ☐ Very hesitant

21. In your opinion, how hesitant are your co-worker(s) to care for :

a. People living with or thought to be living with HIV ?

☐ Not hesitant ☐ A little hesitant ☐ Somewhat hesitant ☐ Very hesitant

b. Men who have sex with men or thought to be having sex with men?

☐ Not hesitant ☐ A little hesitant ☐ Somewhat hesitant ☐ Very hesitant

c. Sex workers or people thought to be sex workers?

☐ Not hesitant ☐ A little hesitant ☐ Somewhat hesitant ☐ Very hesitant

d. Sexually active adolescents/youth or thought to be sexually active adolescents/youth?

☐ Not hesitant ☐ A little hesitant ☐ Somewhat hesitant ☐ Very hesitant

e. People who inject drugs for non-medical reasons or thought to inject drugs?

☐ Not hesitant ☐ A little hesitant ☐ Somewhat hesitant ☐ Very hesitant

SECTION 4: HEALTH FACILITY POLICIES

Now we are going to ask about the institutional policy and work environment in your facility.

22. In my facility nobody allowed to test a patient for HIV without his/her knowledge.

☐ Yes ☐ No ☐ Don't Know

23. I will get into trouble at work if I discriminate against persons living with HIV.

☐ Yes ☐ No ☐ Don't Know

24. I will get into trouble at work if I discriminate against patients who are men who have sex with men.

☐ Yes ☐ No ☐ Don't Know

25. I will get into trouble at work if I discriminate against patients who are sex workers

☐ Yes ☐ No ☐ Don't Know

26. I will get into trouble at work if I discriminate against a sexually active adolescent/youth.

☐ Yes ☐ No ☐ Don't Know

27. I will get into trouble at work if I discriminate against patients who inject drugs for non-medical reasons.

☐ Yes ☐ No ☐ Don't Know

28. Do you strongly agree, agree, disagree, or strongly disagree with the following statements?

a. There are adequate supplies (personal protective equipment (PPE)) in my health facility that reduce my risk of becoming infected with HIV.

☐ Strongly Agree ☐ Agree ☐ Disagree ☐ Strongly Disagree

b. There are standardized procedures/protocols in my health facility that reduce my risk of becoming infected with HIV.

☐ Strongly Agree ☐ Agree ☐ Disagree ☐ Strongly Disagree

29. My health facility has written guidelines to protect persons living with HIV from discrimination.

☐ Yes ☐ No ☐ Don't Know

30. My health facility has written guidelines to protect patients who are men who have sex with men from discrimination.

☐ Yes ☐ No ☐ Don't Know

31. My health facility has written guidelines to protect patients who are sex workers from discrimination.

☐ Yes ☐ No ☐ Don't Know

32. Does your facility have post-exposure prophylactic (PEP) protocol placed where all staff can see?

☐ Yes ☐ No

33. Do you have access to post-exposure prophylactic (PEP) medications in your health facility?

☐ Yes ☐ No ☐ Don't Know

SECTION 5: OPINIONS ABOUT PEOPLE LIVING WITH HIV AND OTHER KEY POPULATIONS.

Now we are going to ask about opinions related to people living with HIV and other key populations.

34. Do you strongly agree, agree, disagree, or strongly disagree with the following statements?

a. Most people living with HIV do not care if they infect other people.
☐ Strongly Agree ☐ Agree ☐ Disagree ☐ Strongly Disagree

b. People living with HIV should feel ashamed of themselves.
☐ Strongly Agree ☐ Agree ☐ Disagree ☐ Strongly Disagree

c. Most people living with HIV have had many sexual partners.
☐ Strongly Agree ☐ Agree ☐ Disagree ☐ Strongly Disagree

d. Most female sex workers are living with HIV
☐ Strongly Agree ☐ Agree ☐ Disagree ☐ Strongly Disagree

e. People get infected with HIV because they engage in irresponsible behaviors.
☐ Strongly Agree ☐ Agree ☐ Disagree ☐ Strongly Disagree

f. HIV is punishment for bad behavior
☐ Strongly Agree ☐ Agree ☐ Disagree ☐ Strongly Disagree

g. Most men living with HIV are men who have sex with other men
☐ Strongly Agree ☐ Agree ☐ Disagree ☐ Strongly Disagree

h. Men who have sex with men were born that way.
☐ Strongly Agree ☐ Agree ☐ Disagree ☐ Strongly Disagree

i. Women living with HIV should be allowed to have babies if they wish.
☐ Strongly Agree ☐ Agree ☐ Disagree ☐ Strongly Disagree

j. I would be ashamed if I had HIV.
☐ Strongly Agree ☐ Agree ☐ Disagree ☐ Strongly Disagree

k. I would be ashamed if someone in my family had HIV.
☐ Strongly Agree ☐ Agree ☐ Disagree ☐ Strongly Disagree

l. A woman living with HIV should be provided treatment only if she is using family planning methods.
☐ Strongly Agree ☐ Agree ☐ Disagree ☐ Strongly Disagree

35. Do you strongly agree, agree, disagree, or strongly disagree with the following statements?

a. If I had a choice, I would prefer not to provide services to men who have sex with men.
- ☐ Strongly Agree
- ☐ Agree
- ☐ Disagree
- ☐ Strongly Disagree

b. If I had a choice, I would prefer not to provide services to sex workers
- ☐ Strongly Agree
- ☐ Agree
- ☐ Disagree
- ☐ Strongly Disagree

c. If I had a choice, I would prefer not to provide HIV and other sexual and reproductive health (SRH) services to sexually active adolescents.
- ☐ Strongly Agree
- ☐ Agree
- ☐ Disagree
- ☐ Strongly Disagree

d. It should be optional for healthcare workers to care for key populations (MSM, sex workers and drug users).
- ☐ Strongly Agree
- ☐ Agree
- ☐ Disagree
- ☐ Strongly Disagree

36. Compared to this time last year, do you think there has been a change in behavior towards clients living with HIV in this facility?

☐ Much worse ☐ A little worse ☐ No change ☐ A little better ☐ Much better

37. Do you provide any services (medical or non-medical) to pregnant women or do any work in the following departments: antenatal care, prevention of mother to child transmission, labor or delivery wards, or postpartum care?

☐ Yes **CONTINUE ON TO SECTION 6, Q37 (next page).**

☐ No **YOU HAVE COMPLETED THE QUESTIONNAIRE. THANK YOU FOR SHARING YOUR VALUABLE TIME AND INSIGHTS WITH US.**

SECTION 6: ANTENATAL CARE, PREVENTION OF MOTHER-TO-CHILD TRANSMISSION, LABOR AND DELIVERY WARDS

The following section is to be completed by any staff who works in the following departments: antenatal care, prevention of mother-to-child transmission of HIV, labor and delivery rooms and post-natal care.

38. How worried are you about assisting in labor and delivery

a. If the woman is living with HIV?
☐ Not worried ☐ A little worried ☐ Worried ☐ Very worried ☐ Not applicable

b. If the woman had been on anti-retroviral (ARV) treatment for six months or more
☐ Not worried ☐ A little worried ☐ Worried ☐ Very worried ☐ Not applicable

c. If the woman has been on anti-retroviral (ARV) treatment for less than a month or not at all
☐ Not worried ☐ A little worried ☐ Worried ☐ Very worried ☐ Not applicable

39. ***In the past 6 months***, how often have you observed other healthcare providers:

a) Performing an HIV test on a pregnant woman without her informed consent during ANC?
☐ Never ☐ Once or twice ☐ Several times ☐ Most of the time

b) Performing an HIV test on a pregnant woman without her informed consent during labor and delivery?
☐ Never ☐ Once or twice ☐ Several times ☐ Most of the time

c) Neglecting a woman living with HIV during labor and delivery because of her HIV status?
☐ Never ☐ Once or twice ☐ Several times ☐ Most of the time

d) Using additional infection-control procedures (e.g., double gloves) with a pregnant woman living with HIV during labor and delivery because of her HIV status?

☐ Never ☐ Once or twice ☐ Several times ☐ Most of the time

e) Disclosing the status of a pregnant woman living with HIV to others without her consent?

☐ Never ☐ Once or twice ☐ Several times ☐ Most of the time

40. Do you strongly agree, agree, disagree, or strongly disagree with the following statements?

a) If a pregnant woman is HIV positive, her partner/child's father has a right to know.
☐ Strongly Agree ☐ Agree ☐ Disagree ☐ Strongly Disagree

b) Pregnant women who refuse HIV testing are irresponsible.
☐ Strongly Agree ☐ Agree ☐ Disagree ☐ Strongly Disagree

c) Women living with HIV should not get pregnant if they already have children.
☐ Strongly Agree ☐ Agree ☐ Disagree ☐ Strongly Disagree

d) It can be appropriate to sterilize a woman living with HIV, even if this is not her choice.
☐ Strongly Agree ☐ Agree ☐ Disagree ☐ Strongly Disagree

e) Women living with HIV should not breastfeed their babies
☐ Strongly Agree ☐ Agree ☐ Disagree ☐ Strongly Disagree

Thank You

Start Time:

Date of data Collection:

Name of Interviewer:

Name of Facility:

Facility Code:

Participation Identification Code (PID) Number:

Field Supervisor:

UNDERSTANDING HIV RELATED STIGMA AND DISCRIMINATION AMONG HEALTH FACILITY STAFF

ENGLISH

SECTION 1: BACKGROUND INFORMATION

First we would like to ask about your background.

1. Please state your age from the following ranges: ☐ 18-24 ☐ 25-34 ☐ 35-44 ☐ 45-54 ☐ 55 and over

2. What is your sex?

   ☐ Female ☐ Male

3. What is the highest level of school you completed?

   ☐ None ☐ Primary/Basic/Middle School ☐ Technical/Vocational ☐ Secondary ☐ Tertiary

   ☐ Other: ________

4. What is your current job in this facility **(<u>the job you spend the majority of your time on</u>)**?

   ☐ Accountant/Cashier/Revenue Collector ☐ Cleaning Staff/Orderly

   ☐ Dentist ☐ Dental Technician/Hygienist ☐ Doctor

   ☐ Nurse Assistant ☐ Nurse ☐Midwife ☐ Receptionist

   ☐ Physician Assistant ☐ Medical Records Personnel ☐ Pharmacist

   ☐ Pharmacist Technologist ☐ Biomedical Scientist/Laboratory Technician

   ☐ Peer Educator /Model of Hope ☐ Security Staff

   ☐ Other: ________

5. How many years have you been working at this facility? ____ Years ____ Months

6. Have you ever worked in a department that specializes in offering services in HIV care and treatment?

   ☐Yes ☐ No

7. ***In the past one month***, approximately how many HIV-positive people have you personally provided with care or services? ☐☐☐

8. Did you ever receive training (either formal or in workshops) in the following subjects? (Check all that apply.)

		Yes	No
a.	Infection control and universal precautions (including post-exposure prophylaxis - PEP)	☐	☐
b.	Patients’ informed consent, privacy, and confidentiality	☐	☐
c.	HIV stigma and discrimination	☐	☐
d.	Key population stigma and discrimination	☐	☐

SECTION 2: INFECTION CONTROL

Now we will ask you about HIV infection concerns in your health facility.

9. How worried would you be about getting HIV if you did the following?
If any of the following is not one of your job responsibilities, please select "Not applicable."

i. Touched the clothing or bedding of a person living with HIV
☐ Not worried ☐ A little worried ☐ Worried ☐ Very worried ☐ Not applicable

ii. Dressed the wounds of a person living with HIV
☐ Not worried ☐ A little worried ☐ Worried ☐ Very worried ☐ Not applicable

iii. Drew blood from a person living with HIV
☐ Not worried ☐ A little worried ☐ Worried ☐ Very worried ☐ Not applicable

iv. Took the temperature, blood pressure or pulse of a patient living with HIV
☐ Not worried ☐ A little worried ☐ Worried ☐ Very worried ☐ Not applicable

10. Do you usually use any of the following measures when providing care or services for a person living with HIV? ***If any of the following are not applicable to your job responsibilities, please select "Not applicable."***

a. Avoid physical contact
☐ Yes ☐ No ☐ Not applicable

b. Wear double gloves
☐ Yes ☐ No ☐ Not applicable

c. Wear gloves during all aspects of the patient's care
☐ Yes ☐ No ☐ Not applicable

d. Use any extra precautionary measures with persons living with HIV that you do not use with other patients
☐ Yes ☐ No ☐ Not applicable

SECTION 3: HEALTH FACILITY ENVIRONMENT

Now we will ask about practices in your health facility and your experiences working in a facility that provides care to people living with HIV and other key populations.

11. In the past 6 months, how often have you observed healthcare workers unwilling to care for:

a. Persons living with or thought to be living with HIV?
☐ Never ☐ Once or twice ☐ Several times ☐ Most of the time

b. Men who have sex with men or thought to be having sex with men?
☐ Never ☐ Once or twice ☐ Several times ☐ Most of the time

c. A sex worker or people thought to be sex workers?
☐ Never ☐ Once or twice ☐ Several times ☐ Most of the time

d. A sexually active adolescent/youth or thought to be sexually active adolescent/youth?
☐ Never ☐ Once or twice ☐ Several times ☐ Most of the time

e. A person who injects drugs for non-medical reasons or thought to be injecting drugs?
☐ Never ☐ Once or twice ☐ Several times ☐ Most of the time

12. In the past 6 months, how often have you observed healthcare workers providing poorer quality of care for:

a. Persons living with or thought to be living with HIV?
☐ Never ☐ Once or twice ☐ Several times ☐ Most of the time

b. Men who have sex with men or thought to be having sex with men?
☐ Never ☐ Once or twice ☐ Several times ☐ Most of the time

c. A sex worker or people thought to be sex workers?
☐ Never ☐ Once or twice ☐ Several times ☐ Most of the time

d. A sexually active adolescent/youth or thought to be a sexually active adolescent/youth?
☐ Never ☐ Once or twice ☐ Several times ☐ Most of the time

e. A person who injects drugs for non-medical reasons or thought to be injecting drugs?
☐ Never ☐ Once or twice ☐ Several times ☐ Most of the time

13. In the past 6 months, how often have you observed healthcare workers talking badly about:

a. Persons living with or thought to be living with HIV?
☐ Never ☐ Once or twice ☐ Several times ☐ Most of the time

b. Men who have sex with men or thought to be having sex with men?
☐ Never ☐ Once or twice ☐ Several times ☐ Most of the time

c. A sex worker or people thought to be sex workers?
☐ Never ☐ Once or twice ☐ Several times ☐ Most of the time

d. A sexually active adolescent/youth or thought to be sexually active adolescent/youth?
☐ Never ☐ Once or twice ☐ Several times ☐ Most of the time

e. A person who injects drugs for non-medical reasons or thought to be injecting drugs for non-medical reasons?
☐ Never ☐ Once or twice ☐ Several times ☐ Most of the time

14. In the past 6 months, how often have you observed healthcare workers disclosing a person living with HIV or other status without their consent, in a situation that was not medically required?
☐ Never ☐ Once or twice ☐ Several times ☐ Most of the time

15. How worried are you about:

a. Friends and family treating you differently because you care for persons living with HIV?
☐Never worried ☐Worried ☐Sometimes worried ☐Always worried ☐Not applicable

b. Friends and family treating you differently because you care for persons who are men who have sex with men?
☐Never worried ☐Worried ☐Sometimes worried ☐Always worried ☐Not applicable

16. In your opinion, how hesitant are healthcare workers to take an HIV test, in this facility, due to fear of other people's reactions if the test is positive?

☐ Not hesitant ☐ A little hesitant ☐ Somewhat hesitant ☐ Very hesitant

17. How hesitant are you to take an HIV test, in this facility, due to fear of other people's reactions if the test is positive?

☐ Not hesitant ☐ A little hesitant ☐ Somewhat hesitant ☐ Very hesitant

18. In your opinion, how hesitant are healthcare workers in this facility to work alongside a co-worker living with HIV, regardless of their duties?
☐ Not hesitant ☐ A little hesitant ☐ Somewhat hesitant ☐ Very hesitant

19. How confident are you that when you take an HIV test in this facility the results will be confidential?
☐ Not confident ☐ A little confident ☐ Somewhat confident ☐ Very Confident

20. In your opinion, how hesitant do you think a healthcare worker living with HIV would be to seek healthcare in this facility?

☐ Not hesitant ☐ A little hesitant ☐ Somewhat hesitant ☐ Very hesitant

21. In your opinion, how hesitant are your co-worker(s) to care for :

a. People living with or thought to be living with HIV ?

☐ Not hesitant ☐ A little hesitant ☐ Somewhat hesitant ☐ Very hesitant

b. Men who have sex with men or thought to be having sex with men?

☐ Not hesitant ☐ A little hesitant ☐ Somewhat hesitant ☐ Very hesitant

c. Sex workers or people thought to be sex workers?

☐ Not hesitant ☐ A little hesitant ☐ Somewhat hesitant ☐ Very hesitant

d. Sexually active adolescents/youth or thought to be sexually active adolescents/youth?

☐ Not hesitant ☐ A little hesitant ☐ Somewhat hesitant ☐ Very hesitant

e. People who inject drugs for non-medical reasons or thought to inject drugs?

☐ Not hesitant ☐ A little hesitant ☐ Somewhat hesitant ☐ Very hesitant

SECTION 4: HEALTH FACILITY POLICIES

Now we are going to ask about the institutional policy and work environment in your facility.

22. In my facility nobody allowed to test a patient for HIV without his/her knowledge.
☐ Yes ☐ No ☐ Don't Know

23. I will get into trouble at work if I discriminate against persons living with HIV.
☐ Yes ☐ No ☐ Don't Know

24. I will get into trouble at work if I discriminate against patients who are men who have sex with men.
☐ Yes ☐ No ☐ Don't Know

25. I will get into trouble at work if I discriminate against patients who are sex workers
☐ Yes ☐ No ☐ Don't Know

26. I will get into trouble at work if I discriminate against a sexually active adolescent/youth.
☐ Yes ☐ No ☐ Don't Know

27. I will get into trouble at work if I discriminate against patients who inject drugs for non-medical reasons.

☐ Yes ☐ No ☐ Don't Know

28. Do you strongly agree, agree, disagree, or strongly disagree with the following statements?

a. There are adequate supplies (personal protective equipment (PPE)) in my health facility that reduce my risk of becoming infected with HIV.
☐ Strongly Agree ☐ Agree ☐ Disagree ☐ Strongly Disagree

b. There are standardized procedures/protocols in my health facility that reduce my risk of becoming infected with HIV.

☐ Strongly Agree ☐ Agree ☐ Disagree ☐ Strongly Disagree

29. My health facility has written guidelines to protect persons living with HIV from discrimination.
☐ Yes ☐ No ☐ Don't Know

30. My health facility has written guidelines to protect patients who are men who have sex with men from discrimination.
☐ Yes ☐ No ☐ Don't Know

31. My health facility has written guidelines to protect patients who are sex workers from discrimination.
☐ Yes ☐ No ☐ Don't Know

32. Does your facility have post-exposure prophylactic (PEP) protocol placed where all staff can see?
☐ Yes ☐ No

33. Do you have access to post-exposure prophylactic (PEP) medications in your health facility?
☐ Yes ☐ No ☐ Don't Know

SECTION 5: OPINIONS ABOUT PEOPLE LIVING WITH HIV AND OTHER KEY POPULATIONS.

Now we are going to ask about opinions related to people living with HIV and other key populations.

34. Do you strongly agree, agree, disagree, or strongly disagree with the following statements?

a. Most people living with HIV do not care if they infect other people.
☐ Strongly Agree ☐ Agree ☐ Disagree ☐ Strongly Disagree

b. People living with HIV should feel ashamed of themselves.
☐ Strongly Agree ☐ Agree ☐ Disagree ☐ Strongly Disagree

c. Most people living with HIV have had many sexual partners.
☐ Strongly Agree ☐ Agree ☐ Disagree ☐ Strongly Disagree

d. Most female sex workers are living with HIV
☐ Strongly Agree ☐ Agree ☐ Disagree ☐ Strongly Disagree

e. People get infected with HIV because they engage in irresponsible behaviors.
☐ Strongly Agree ☐ Agree ☐ Disagree ☐ Strongly Disagree

f. HIV is punishment for bad behavior
☐ Strongly Agree ☐ Agree ☐ Disagree ☐ Strongly Disagree

g. Most men living with HIV are men who have sex with other men
☐ Strongly Agree ☐ Agree ☐ Disagree ☐ Strongly Disagree

h. Men who have sex with men were born that way.
☐ Strongly Agree ☐ Agree ☐ Disagree ☐ Strongly Disagree

i. Women living with HIV should be allowed to have babies if they wish.
☐ Strongly Agree ☐ Agree ☐ Disagree ☐ Strongly Disagree

j. I would be ashamed if I had HIV.
☐ Strongly Agree ☐ Agree ☐ Disagree ☐ Strongly Disagree

k. I would be ashamed if someone in my family had HIV.
☐ Strongly Agree ☐ Agree ☐ Disagree ☐ Strongly Disagree

l. A woman living with HIV should be provided treatment only if she is using family planning methods.
☐ Strongly Agree ☐ Agree ☐ Disagree ☐ Strongly Disagree

35. Do you strongly agree, agree, disagree, or strongly disagree with the following statements?

a. If I had a choice, I would prefer not to provide services to men who have sex with men.
- ☐ Strongly Agree
- ☐ Agree
- ☐ Disagree
- ☐ Strongly Disagree

b. If I had a choice, I would prefer not to provide services to sex workers
- ☐ Strongly Agree
- ☐ Agree
- ☐ Disagree
- ☐ Strongly Disagree

c. If I had a choice, I would prefer not to provide HIV and other sexual and reproductive health (SRH) services to sexually active adolescents.
- ☐ Strongly Agree
- ☐ Agree
- ☐ Disagree
- ☐ Strongly Disagree

d. It should be optional for healthcare workers to care for key populations (MSM, sex workers and drug users).
- ☐ Strongly Agree
- ☐ Agree
- ☐ Disagree
- ☐ Strongly Disagree

36. Do you provide any services (medical or non-medical) to pregnant women or do any work in the following departments: antenatal care, prevention of mother to child transmission, labor or delivery wards, or postpartum care?

☐ Yes **CONTINUE ON TO SECTION 6, Q. 36 (next page).**

☐ No **YOU HAVE COMPLETED THE QUESTIONNAIRE. THANK YOU FOR SHARING YOUR VALUABLE TIME AND INSIGHTS WITH US.**

SECTION 6: ANTENATAL CARE, PREVENTION OF MOTHER-TO-CHILD TRANSMISSION, LABOR AND DELIVERY WARDS

The following section is to be completed by any staff who works in the following departments: antenatal care, prevention of mother-to-child transmission of HIV, labor and delivery rooms and post-natal care.

37. How worried are you about assisting in labor and delivery

a. If the woman is living with HIV?
☐ Not worried ☐ A little worried ☐ Worried ☐ Very worried ☐ Not applicable

b. If the woman had been on anti-retroviral (ARV) treatment for six months or more
☐ Not worried ☐ A little worried ☐ Worried ☐ Very worried ☐ Not applicable

c. If the woman has been on anti-retroviral (ARV) treatment for less than a month or not at all
☐ Not worried ☐ A little worried ☐ Worried ☐ Very worried ☐ Not applicable

38. ***In the past 6 months***, how often have you observed other healthcare providers:

a) Performing an HIV test on a pregnant woman without her informed consent during ANC?
☐ Never ☐ Once or twice ☐ Several times ☐ Most of the time

b) Performing an HIV test on a pregnant woman without her informed consent during labor and delivery?
☐ Never ☐ Once or twice ☐ Several times ☐ Most of the time

c) Neglecting a woman living with HIV during labor and delivery because of her HIV status?
☐ Never ☐ Once or twice ☐ Several times ☐ Most of the time

d) Using additional infection-control procedures (e.g., double gloves) with a pregnant woman living with HIV during labor and delivery because of her HIV status?
☐ Never ☐ Once or twice ☐ Several times ☐ Most of the time

e) Disclosing the status of a pregnant woman living with HIV to others without her consent?
☐ Never ☐ Once or twice ☐ Several times ☐ Most of the time

39. Do you strongly agree, agree, disagree, or strongly disagree with the following statements?

a) If a pregnant woman is HIV positive, her partner/child's father has a right to know.
☐ Strongly Agree ☐ Agree ☐ Disagree ☐ Strongly Disagree

b) Pregnant women who refuse HIV testing are irresponsible.
☐ Strongly Agree ☐ Agree ☐ Disagree ☐ Strongly Disagree

c) Women living with HIV should not get pregnant if they already have children.
☐ Strongly Agree ☐ Agree ☐ Disagree ☐ Strongly Disagree

d) It can be appropriate to sterilize a woman living with HIV, even if this is not her choice.
☐ Strongly Agree ☐ Agree ☐ Disagree ☐ Strongly Disagree

e) Women living with HIV should not breastfeed their babies
☐ Strongly Agree ☐ Agree ☐ Disagree ☐ Strongly Disagree

End Time:

Thank You
