# Supplementary material for: Results from a difference‐in‐differences evaluation of health facility HIV and key population stigma‐reduction interventions in Ghana
Source: J Int AIDS Soc. 2020 Apr 23;23(4):e25483. doi: 10.1002/jia2.25483 (PMC7180216; doi:10.1002/jia2.25483)
Supplement: Supplementary file 2 — Data S2. Inverse probability of treatment weighting models. [file JIA2-23-e25483-s002.docx]

**Supplement 2. Inverse Probability of Treatment Weighting Models**

As described in the main text, this study was designed as a comparison between intervention facilities and comparison facilities, with outcomes measured before and after implementation began. Treatment effects are estimated as differences-in-differences in the probability of each outcome.

This design is robust to most potential causes of confounding. It accounts for other causes of the outcomes of interest if they (a) are time-invariant or (b) change equivalently from before-to-after intervention in both groups. If, however, other causes of the outcome of interest change differently across the treatment and comparison group, difference-in-differences estimates may be confounded. The standard way to guard against this risk is to take multiple pre-intervention outcome measurements; if other determinants of an outcome were constant or changing equivalently in both groups, pre-intervention trends in the outcome should be parallel (1).

In this study, we could not take multiple pre-intervention measurements for practical (funding timetables precluded it) and ethical (documentation of high levels of stigma demanded action) reasons. We have no reason to expect that there existed systematically different trends over time in the intervention and comparison group, but we cannot directly rule that out. Instead, we seek to balance potential determinants of stigma and discrimination outcomes across the four time-by-intervention groups using inverse probability of treatment weighting (IPTW) (2). To the extent the variables in the IPTW models are determinants—or proxies for determinants—of difference-in-differences confounders, the weighted treatment effect estimates should have less risk of confounding.

To construct IPT weights, we followed the approach of White *et al*. (3), which is a slight modification of Stuart *et al* (2). (Weights are constructed such that each group aims to mirror the collective sample across the four groups, rather than the pre-intervention intervention group. This results in an estimate of average treatment effects rather than average treatment effects among the treated.) As a first step, we modeled propensity for an observation to be each group as a function of covariates described in more detail below using multinomial logistic regression. We then constructed stabilized IPT weights. These weights equal, for each observation, the proportion of the sample in the time-by-treatment group the observation was actually in divided by the observations estimated propensity to be in the group it was actually in (4).

We included the following covariates in the propensity score models, all as indicator variables: age (collected in as 18-24, 10-year intervals from 25 to 54 years old, and 55+), region, sex, staff category (senior medical, other medical, and administrative or support staff), tenure at the facility (<2 years, 2-5 years, 5+years), whether the respondent has ever worked in an HIV-specific department, and quintiles of the number of persons living with HIV (PLHIV) the respondent reports personally providing care in the past month. For three variables, missing responses were coded as an additional category: time working at the facility, experience working in an HIV-specific department, and number of PLHIV treated in the last month. Other covariates had too few missing responses models to be stable if included as a separate category. Observations with missing values for those responses were excluded from the IPTW-adjusted models (though not from the unweighted difference-in-differences models), causing 85 (3.7%) of observations to be excluded.

We fit separate propensity score models, and calculated separate IPT weights, for the full sample and the clinician-only subsample.

We assessed covariate balance using standardized differences and variance ratios for each time-by-intervention group compared to the pre-intervention period for the intervention group. All balance diagnostics were calculated without the actual outcome variable in the models to avoid the risk that propensity score models would be refit until favorable results were obtained. As detailed in the tables below, all standardized differences and variance ratios meet common guidelines for standardized differences to be between -0.25 and 0.25 and variance ratios to be between 0.5 and 2.0 (5). More stringent guidelines for standardized differences to be between -0.1 and 0.1 were met for virtually all covariates (6). Given the number of covariates and four treatment categories, some mild imbalance in a few covariates is to be expected (7).

**Table 1. Standardized differences and variance ratios for IPTW model in the full sample.**

| **Comparison, pre vs. intervention, pre** | **Standardized diff.** | | **Variance ratio** | |
| --- | --- | --- | --- | --- |
|  | Raw | Weighted | Raw | Weighted |
| Age |  |  |  |  |
| 25-34 | .1040252 | .0122487 | 1.015398 | 1.001968 |
| 35-44 | .0043363 | -.0278535 | 1.005499 | .9749464 |
| 45-54 | -.0917568 | -.0119291 | .7817989 | .9684408 |
| 55+ | .0268488 | .0147548 | 1.087755 | 1.052578 |
| Male sex | -.0560286 | .0072631 | .9703604 | 1.004588 |
| Staff category |  |  |  |  |
| Mid-level medical | -.0557341 | -.0386751 | 1.069954 | 1.048624 |
| Administrative & support | .1121051 | .0213394 | 1.199116 | 1.036951 |
| Time working at facility |  |  |  |  |
| 2 to <5 years | .0679598 | -.0025371 | 1.065029 | .9979716 |
| 5+ years | -.081259 | -.0065941 | .9733476 | .9980697 |
| Missing | -.0822747 | .0254877 | .6906696 | 1.148803 |
| Experience working in a clinic specializing in HIV services |  |  |  |  |
| Yes | -.1777425 | -.0116064 | .9683559 | .9977206 |
| Missing | .0050294 | .0256962 | 1.023782 | 1.138041 |
| Quintiles of number of PLHIV provided care |  |  |  |  |
| Second | .1465448 | .0094528 | 1.398025 | 1.02186 |
| Middle | .1027724 | -.0022589 | 1.181067 | .9968426 |
| Fourth | -.1187521 | .0048063 | .7332012 | 1.013094 |
| Highest | -.2548804 | .0009321 | .6017416 | 1.002001 |
| No response | .1643907 | -.0864767 | 1.367124 | .8882729 |
| Region |  |  |  |  |
| Brong Ahafo | -.1770742 | .0246299 | .7083902 | 1.054564 |
| Eastern | .1111302 | -.0334588 | 1.170678 | .9547115 |
| Greater Accra | -.0836678 | .1332382 | .9185519 | 1.172384 |
| Western | -.010789 | -.0251572 | .979622 | .9579002 |
|  |  | |  | |
| **Comparison, post vs. intervention, pre** | **Standardized diff.** | | **Variance ratio** | |
|  | Raw | Weighted | Raw | Weighted |
| Age |  |  |  |  |
| 25-34 | -.0104604 | .0001692 | .9974143 | 1.000108 |
| 35-44 | .293486 | -.0313047 | 1.291419 | .9717448 |
| 45-54 | -.0619864 | .0186503 | .8514107 | 1.049986 |
| 55+ | -.0579033 | .01686 | .8191575 | 1.060137 |
| Male sex | -.1854145 | .0023834 | .8815082 | 1.001569 |
| Staff category |  |  |  |  |
| Mid-level medical | .0314604 | -.0303644 | .9590595 | 1.038354 |
| Administrative & support | -.0351046 | .017624 | .9364097 | 1.030547 |
| Time working at facility |  |  |  |  |
| 2 to <5 years | .0215316 | -.0226484 | 1.021521 | .9808505 |
| 5+ years | .039142 | .0132526 | 1.008446 | 1.003871 |
| Missing | -.1113399 | -.0009561 | .5914758 | .994679 |
| Experience working in a clinic specializing in HIV services |  |  |  |  |
| Yes | -.1373042 | .0249519 | .9809589 | 1.004255 |
| Missing | .0167584 | .0543541 | 1.079853 | 1.300953 |
| Quintiles of number of PLHIV provided care |  |  |  |  |
| Second | .091591 | .0079114 | 1.24705 | 1.018301 |
| Middle | .1454252 | .0067149 | 1.25311 | 1.009687 |
| Fourth | -.0826137 | .0190658 | .8127481 | 1.051901 |
| Highest | -.1214695 | .0379437 | .8112752 | 1.078127 |
| No response | .2767322 | -.0635939 | 1.604903 | .9185559 |
| Region |  |  |  |  |
| Brong Ahafo | -.167138 | .0495122 | .7247707 | 1.109739 |
| Eastern | .0904284 | .0134106 | 1.140032 | 1.01793 |
| Greater Accra | -.0813576 | .0673603 | .9209512 | 1.090357 |
| Western | .0250943 | -.0338295 | 1.04773 | .9432843 |
|  |  |  |  |  |
| **Intervention, post vs. intervention, pre** | **Standardized diff.** | | **Variance ratio** | |
|  | Raw | Weighted | Raw | Weighted |
| Age |  |  |  |  |
| 25-34 | .0434651 | -.0281659 | 1.008964 | .994587 |
| 35-44 | .1378237 | -.0068234 | 1.157144 | .994004 |
| 45-54 | .0445488 | .0241286 | 1.109178 | 1.064705 |
| 55+ | -.1247439 | .007215 | .6250455 | 1.025604 |
| Male sex | -.0702821 | .0244675 | .9617143 | 1.014833 |
| Staff category |  |  |  |  |
| Mid-level medical | -.0364395 | -.0447269 | 1.046052 | 1.056001 |
| Administrative & support | .0069998 | .0372849 | 1.012599 | 1.064272 |
| Time working at facility |  |  |  |  |
| 2 to <5 years | .0969718 | -.0312987 | 1.090106 | .9732604 |
| 5+ years | -.0245158 | -.0021407 | .9931988 | .9994045 |
| Missing | -.232051 | .0654829 | .234713 | 1.400634 |
| Experience working in a clinic specializing in HIV services |  |  |  |  |
| Yes | -.0421517 | .0059228 | .9980121 | 1.001152 |
| Missing | -.0734778 | .0052834 | .6801988 | 1.027786 |
| Quintiles of number of PLHIV provided care |  |  |  |  |
| Second | .0267203 | .0060536 | 1.070978 | 1.013987 |
| Middle | .1990522 | .0010332 | 1.33955 | 1.00153 |
| Fourth | -.0453134 | .0146529 | .8963547 | 1.039824 |
| Highest | -.1989307 | .0064285 | .6890002 | 1.013272 |
| No response | .3583505 | -.0989128 | 1.761711 | .8716108 |
| Region |  |  |  |  |
| Brong Ahafo | .0176783 | -.0281659 | 1.028432 | 1.06045 |
| Eastern | -.0260085 | -.0068234 | .9583831 | .9876097 |
| Greater Accra | .0068833 | .0241286 | 1.006185 | 1.09249 |
| Western | .0033763 | .007215 | 1.006348 | .9434013 |

**Table 2. Standardized differences and variance ratios for IPTW model in the clinical staff sample.**

| **Comparison, pre vs. intervention, pre** | **Standardized diff.** | | **Variance ratio** | |
| --- | --- | --- | --- | --- |
|  | Raw | Weighted | Raw | Weighted |
| Age |  |  |  |  |
| 25-34 | .1590102 | .0043846 | .9983696 | 1.000073 |
| 35-44 | -.0054128 | -.0270596 | .9935476 | .9750732 |
| 45-54 | -.1203484 | -.0139304 | .6681694 | .9559131 |
| 55+ | -.0506174 | .0383585 | .8331596 | 1.166978 |
| Male sex | -.0769418 | .0104893 | .9385933 | 1.009786 |
| Staff category |  |  |  |  |
| Mid-level medical | .0826303 | -.0458557 | .7498436 | 1.159299 |
| Time working at facility |  |  |  |  |
| 2 to <5 years | .0790779 | .007888 | 1.074261 | 1.006318 |
| 5+ years | -.0789597 | -.0118579 | .9664236 | .9952452 |
| Missing | -.0880641 | .0213925 | .6832829 | 1.119555 |
| Experience working in a clinic specializing in HIV services |  |  |  |  |
| Yes | -.1866396 | .0218934 | .9960581 | 1.000893 |
| Missing | -.0185964 | .0137736 | .9188626 | 1.072445 |
| Quintiles of number of PLHIV provided care |  |  |  |  |
| Second | .1522675 | .0107777 | 1.378597 | 1.022961 |
| Middle | .1236116 | .0109924 | 1.189608 | 1.013708 |
| Fourth | -.0955499 | .0168237 | .7927274 | 1.043091 |
| Highest | -.2477699 | .0062048 | .6305251 | 1.012179 |
| No response | .1135059 | -.1168038 | 1.273663 | .8346375 |
| Region |  |  |  |  |
| Brong Ahafo | -.2294918 | .0216124 | .6209305 | 1.047109 |
| Eastern | .1164354 | -.0386286 | 1.197903 | .9408086 |
| Greater Accra | -.1567252 | .136035 | .8489115 | 1.177114 |
| Western | .0252717 | -.0202543 | 1.046192 | .9670574 |
|  |  | |  | |
| **Comparison, post vs. intervention, pre** | **Standardized diff.** | | **Variance ratio** | |
|  | Raw | Weighted | Raw | Weighted |
| Age |  |  |  |  |
| 25-34 | .0129879 | -.003258 | 1.001886 | 1.000112 |
| 35-44 | .2951469 | -.0223222 | 1.291569 | .979521 |
| 45-54 | -.0352766 | .0257986 | .8988964 | 1.083541 |
| 55+ | -.1162492 | .0232665 | .6320965 | 1.100119 |
| Male sex | -.164013 | -.0022018 | .8583773 | .9980508 |
| Staff category |  |  |  |  |
| Mid-level medical | .0056473 | -.0375328 | .9823303 | 1.129841 |
| Time working at facility |  |  |  |  |
| 2 to <5 years | .0263145 | -.0256726 | 1.025904 | .9792137 |
| 5+ years | .0605037 | .0236784 | 1.017942 | 1.009024 |
| Missing | -.1234322 | .0098498 | .568228 | 1.05436 |
| Experience working in a clinic specializing in HIV services |  |  |  |  |
| Yes | -.160402 | .0280052 | 1.000699 | 1.000918 |
| Missing | -.0186777 | .0666715 | .9183931 | 1.370235 |
| Quintiles of number of PLHIV provided care |  |  |  |  |
| Second | .0768117 | .0035289 | 1.190149 | 1.007578 |
| Middle | .1651699 | .0228278 | 1.248433 | 1.028144 |
| Fourth | -.0770757 | .0311807 | .8321488 | 1.079985 |
| Highest | -.1434862 | .0260617 | .7879207 | 1.050699 |
| No response | .247198 | -.0971279 | 1.590373 | .8631357 |
| Region |  |  |  |  |
| Brong Ahafo | -.1707722 | .0625849 | .7172977 | 1.136325 |
| Eastern | .0530379 | .0032215 | 1.091636 | 1.004996 |
| Greater Accra | -.1091226 | .0594889 | .8981026 | 1.080709 |
| Western | .0292558 | -.0344143 | 1.053262 | .9437877 |
|  |  |  |  |  |
| **Intervention, post vs. intervention, pre** | **Standardized diff.** | | **Variance ratio** | |
|  | Raw | Weighted | Raw | Weighted |
| Age |  |  |  |  |
| 25-34 | .0510854 | -.0408307 | 1.004844 | .9986516 |
| 35-44 | .1163 | .0000898 | 1.134303 | 1.000139 |
| 45-54 | .078419 | .0435591 | 1.234274 | 1.14205 |
| 55+ | -.1257654 | .0226899 | .6043765 | 1.097546 |
| Male sex | -.093506 | .0296492 | .923818 | 1.026914 |
| Staff category |  |  |  |  |
| Mid-level medical | -.0562662 | -.0277621 | 1.182291 | 1.095536 |
| Time working at facility |  |  |  |  |
| 2 to <5 years | .138815 | -.0351289 | 1.12231 | .9712081 |
| 5+ years | -.0189972 | -.0102944 | .9928633 | .9958406 |
| Missing | -.2454197 | .0557867 | .2251216 | 1.323862 |
| Experience working in a clinic specializing in HIV services |  |  |  |  |
| Yes | -.0189116 | .0407629 | 1.002681 | 1.000724 |
| Missing | -.0958226 | .0044804 | .6099774 | 1.023345 |
| Quintiles of number of PLHIV provided care |  |  |  |  |
| Second | .0138796 | .0015631 | 1.033943 | 1.003368 |
| Middle | .2164487 | .0028975 | 1.316641 | 1.003653 |
| Fourth | -.0187512 | .0254244 | .9587097 | 1.065111 |
| Highest | -.1610149 | .0157533 | .7612795 | 1.030658 |
| No response | .3175679 | -.1344274 | 1.746694 | .8089114 |
| Region |  |  |  |  |
| Brong Ahafo | .0959274 | .0296377 | 1.152053 | 1.064514 |
| Eastern | -.0313148 | -.0102479 | .9452132 | .9844324 |
| Greater Accra | -.0483819 | .0521821 | .9568091 | 1.070998 |
| Western | -.0199967 | -.0151482 | .9634515 | .9753548 |

Worked Cited

1. Angrist J, Pischke J. Mostly Harmless Econometrics: An Empiricist’s Companion. 2009. 392 p.

2. Stuart EA, Huskamp HA, Duckworth K, Simmons J, Song Z, Chernew ME, et al. Using propensity scores in difference-in-differences models to estimate the effects of a policy change. Heal Serv Outcomes Res Methodol. 2014;14(4):166–82.

3. White EE, Downey J, Sathananthan V, Kanjee Z, Kenny A, Waters A, et al. A Community Health Worker Intervention to Increase Childhood Disease Treatment Coverage in Rural Liberia: A Controlled Before-and-After Evaluation. Am J Public Health. 2018 Sep;108(9):1252–9.

4. Austin PC, Stuart EA. Moving towards best practice when using inverse probability of treatment weighting (IPTW) using the propensity score to estimate causal treatment effects in observational studies. Stat Med. 2015 Dec 10;34(28):3661–79.

5. Rubin DB. Using Propensity Scores to Help Design Observational Studies : Application to the Tobacco Litigation. Heal Serv Outcomes Res Methodol. 2001;2(3–4):169–88.

6. Austin PC. Balance diagnostics for comparing the distribution of baseline covariates between treatment groups in propensity-score matched samples. Stat Med. 2009 Nov 10;28(25):3083–107.

7. Garrido MM, Kelley AS, Paris J, Roza K, Meier DE, Morrison RS, et al. Methods for constructing and assessing propensity scores. Health Serv Res. 2014 Oct;49(5):1701–20.
