# Supplementary material for: Results from a difference‐in‐differences evaluation of health facility HIV and key population stigma‐reduction interventions in Ghana
Source: J Int AIDS Soc. 2020 Apr 23;23(4):e25483. doi: 10.1002/jia2.25483 (PMC7180216; doi:10.1002/jia2.25483)
Supplement: Supplementary file 4 — Table S4. False discovery rate q‐values that account for assessing multiple, correlated outcomes [file JIA2-23-e25483-s004.docx]

|  | **Full Sample** | | | | **Medical Staff Only** | | | |
| --- | --- | --- | --- | --- | --- | --- | --- | --- |
|  | Unadjusted | | IPTW-adjusted | | Unadjusted | | IPTW-adjusted | |
|  | p-value | q-value | p-value | q-value | p-value | q-value | p-value | q-value |
| Holding 1+ Stigmatizing attitude toward: |  |  |  |  |  |  |  |  |
| PLHIV | .225 | .253 | .437 | .492 | .086 | .105 | .101 | .123 |
| Women living with HIV | .775 | .775 | .708 | .708 | .631 | .631 | .878 | .878 |
| MSM | .111 | .143 | .142 | .185 | .184 | .202 | .212 | .233 |
| Preferring not to serve 1+ key population | .003 | .005 | .006 | .014 | <.001 | <.001 | .009 | .017 |
| Fearing to conduct 1+ care activity | --- | --- | --- | --- | <.001 | .001 | .001 | .003 |
| Perception that facility policies protect PLHIV and key populations from discrimination | <.001 | <.001 | <.001 | <.001 | .<.001 | <.001 | <.001 | <.001 |
| Believing would get into trouble if discriminate against PLHIV and key populations | .094 | .141 | .046 | .083 | .052 | .072 | .035 | .048 |
| Believing that facility IC policies, PPE, PEP policies, and PEP availability are adequate | <.001 | <.001 | <.001 | .001 | .001 | .002 | .001 | .003 |
| Performing care activities in a stigmatizing/discriminatory way | --- | --- | --- | --- | .008 | .013 | .015 | .024 |
| Observing incidents of stigmatizing/discriminatory care by other staff | .003 | .005 | .060 | .090 | .001 | .002 | .009 | .017 |
| Believing conduct toward PLHIV has improved since one year ago | .001 | .003 | .001 | .003 | .002 | .004 | .001 | .003 |
| DID: difference-in-differences. IPTW: inverse probability of treatment weighting. All estimates are constructed such that a positive DID is an improvement (even if the original measure was scaled so that higher numbers were an undesirable outcome). Fear and avoidance outcomes are restricted only to clinical staff because the sizable majority of non-clinical staff answered “not applicable” to the single item applicable to them. | | | | | | | | |

**Supplemental table. False discovery rate q-values that account for assessing multiple, correlated outcomes**
